# Empathy and perspective-taking in literary fiction: a systematic review and meta-analysis of psychological evidence and theoretical insights

**DOI:** 10.3389/fpsyg.2026.1865031

**Published:** 2026-09-25

**Authors:** Liu Chang

**Affiliations:** School of Literature and Journalism & Communication, Qingdao University, Qingdao, Shandong, China

**Keywords:** empathy, fiction reading, narrative engagement, prosocial behaviour, theory of mind

## Abstract

**Background:**

It has been argued over the years that fiction reading can be useful in making people know other people, but the empirical history is spotty and frequently contradictory. Investigations vary in their design, measurements and quality and there is still no clear vision of how powerful those effects are and in what circumstances they manifest themselves.

**Methods:**

A systematic search was performed in PubMed, Scopus, Web of Science, PsycINFO, and ERIC of studies published within the past 20 years (2000–2025) that investigated literary or prosocial reading relative to empathy, perspective-taking, theory of mind, or prosocial behaviour. Twenty-one studies were eligible. Of these, 12 contributed enough statistical information to be included in the meta-analysis, yielding 14 independent effect sizes (standardized mean differences and correlations, transformed to Hedges’ *g* and Fisher’s *z*). Random-effects models were used, and heterogeneity and potential publication bias was examined with standard tests.

**Results:**

When all eligible outcomes were pooled, reading literary or prosocial texts was associated with a small but statistically reliable advantage in social-cognitive performance (overall *g* = 0.23, 95% CI [0.11, 0.35], *z* = 3.78, *p* < 0.001). For cognitive empathy and theory of mind specifically, experimental studies produced a pooled effect of *g* = 0.27, 95% CI [0.10, 0.44], *z* = 3.15, *p* = 0.002 and correlational work showed an association of *r* = 0.20, 95% CI [0.03, 0.36], *z* = 4.75, *p* < 0.001. Across the 21 primary studies, 10 (47.6%) reported positive or beneficial outcomes, 6 (28.6%) reported mediated or conditional effects, 5 (23.8%) reported null or weak effects, and 3 (14.3%) contributed neurocognitive evidence. Because these evidence descriptors were not mutually exclusive, percentages were not intended to form a partition of the study set.

**Conclusion:**

Taken together, the findings suggest that reading literary fiction does not uniformly transform readers’ empathy, but is linked to modest, context-sensitive gains, particularly in perspective-taking and theory of mind. These gains are most visible when readers are engaged with demanding narratives and are invited, by the text or by instructions, to actively simulate characters’ minds, with affective empathy and prosocial responses emerging more selectively under such conditions.

## 1 Introduction

Empathy and perspective-taking have been extensively studied in both psychology and literary theory, but their definitions and operationalizations vary across theoretical traditions and empirical studies. In particular, the boundaries between cognitive empathy, affective empathy, perspective-taking, and related social-cognitive constructs are not always defined consistently across studies. According to modern psychological models, there are two forms of empathy: affective empathy (sharing of the emotional state of another) and cognitive empathy or perspective-taking (inferential attribution of beliefs, intentions, and emotions to others) (Denham, 2024). There are many psychological models of empathy present today that distinguish between affective empathy, the sharing or resonance of someone else’s emotional state, and cognitive empathy, or the inference or reflection of someone else’s beliefs, intentions, etc. (Denham, 2024). From the available evidence, the functional and neural correlates of these components appear partially distinguishable but are not considered to be fully independent systems. Other models view them as processes that unfold sequentially, occur as a dimensionally distributed process, or interact dynamically. In the case of literary reading, the two parts of the empathy might thus act in overlapping and context-dependent ways in lieu of being fully distinct mechanisms. In literary settings, the two aspects usually work together by engaging in narration where readers simultaneously recreate the experiences of the characters and deduce their inner world. This dual processing has seen literary fiction as a possible training standard of social cognition especially in areas where sensitivity to multifaceted human experiences is needed (Nijhof and Willems, 2015; Schwerin et al., 2025; Schwering et al., 2021).

Empathy is traditionally understood as an inherently prosocial and other-oriented capacity, the “bright empathy” framework (Maibom, 2017). However, extensive research has questioned this one-dimensional perspective, demonstrating that empathy can be equally self-oriented, parochial, or even antisocial (Bloom, 2018). Several mechanisms illustrate this complexity. First, personal distress a self-focused empathic response characterized by anxiety when witnessing another’s suffering may motivate helping only incidentally, as a by-product of relieving one’s own discomfort rather than genuine concern for the other (Singer and Klimecki, 2014). Second, parochial empathy is selectively directed toward ingroup members, potentially increasing intergroup bias rather than promoting universal empathy (Vaughn et al., 2018). Third, “sadistic empathy” refers to the paradoxical enjoyment that some readers may derive from another person’s or a character’s suffering, a concept associated with Breithaupt’s theoretical account of the darker dimensions of empathic engagement (Shankar, 2022). Subsequent discussions have examined related counter-empathic and socially selective responses in interpersonal and intergroup contexts (Cikara et al., 2014; Shankar, 2022). Directly relevant to the present review, it was found that affective empathy showed no significant association with justice sensitivity or moral-rule endorsement, while cognitive empathy and empathic concern did, indicating that these components can dissociate entirely rather than merely differ in effect size (Decety and Yoder, 2016). Within this framework, empathy is conceptualized as a multidimensional construct spanning a continuum from automatic emotional contagion to reflective compassion (de Waal, 2008). The same underlying capacity may produce constructive or destructive outcomes depending on context, target, and individual differences. The present review acknowledges this complexity: the “positive effect” classification is defined as movement toward enhanced empathy, theory of mind, or prosocial outcomes as reported in original studies. In the Discussion, however, alternative interpretations are considered, recognizing that increased empathy does not necessarily equate to increased pro-sociality direction and target matter substantially.

There has been a continuing debate on the nature of the relationship between cognitive and affective empathy, with several competing models proposed. The Two-Systems/Dissociable Model posits that partially separate neural and functional networks support cognitive and affective empathy mentalizing networks (medial prefrontal cortex, temporoparietal junction) associated with cognitive empathy, and simulation networks (anterior insula, anterior cingulate cortex) associated with affective empathy (Denham, 2024; Thompson et al., 2022). The Sequential Process Model suggests that these components are linked sequentially, with affective empathy providing the basis for cognitive empathy, or cognitive empathy directing attention to emotional cues (Singer and Klimecki, 2014). The Graded/Continuum Model proposes dimensional alternatives to the dichotomous model, conceptualizing empathy along a continuum from emotional contagion to reflective compassion (de Waal, 2008; Decety and Yoder, 2016). The Dynamic Interaction Model emphasizes context-dependence, with top-down and bottom-up processes modulating each other (Gamble et al., 2024). The cognitive-affective distinction is adopted here as a working classification framework for analytical consistency, acknowledged as one of several theoretical perspectives.

Cognitively, literary fiction is distinguished by other types of narrative by its focus on depth, ambiguity, and interiority of characters. These aspects require proactive inferential processing, motivating readers to fill in narrative gaps in information, and create the elaborate mental images of characters. In this manner, perspective-taking is a dynamic process of decentering in which the readers put aside their own perspective in favor of the fictional agents (Kucirkova, 2019). Yet, just as perspective-taking can be shaped by fictional contexts, real-world evaluative behaviours such as online reviews are also susceptible to environmental and social pressures, with rainy weather and social influence affecting consumer judgments through negative mood and conformity tendency (Yang et al., 2026). Experimental research indicates that this interaction may stimulate neural networks relating to processing of real-world emotions, implying that imagined interactions may enlist processes related to actual social interaction (Denham, 2024). This is the basis of the notion that simulation-based theories of narrative comprehension propose that the reader is not a passive receiver of information but a prolificant reconstructor of experiential states through the use of imagination.

In spite of these theoretical expectations, the evidence concerning the correlation of literary fiction and empathy is quite controversial and is likely to be argued about. Early experimental research found that a short-term exposure to literary fiction had the potential to improve performance on tasks of theory of mind and empathic inference. However, subsequent replication studies have been inconclusive, as other studies have not found significant causal effects (Denham, 2024). Meta-analytical literature, once again, finds small but statistically significant links between fiction exposure and social-cognitive abilities, but these tend to be small and can be influenced by differences in methods (Lenhart and Richter, 2025; Mar et al., 2009). This discrepancy raises important concerns regarding measurement validity, as several widely used measures capture only limited facets of empathy, typically emotion recognition, while failing to assess experiential or moral dimensions of empathic response (Jia et al., 2025; Lyu et al., 2026).

The recent developments have moved towards more sophisticated models which fuse literary theory and cognitive science. Recent research highlights the role of such features of narration as foregrounding, polyphony, and interpretive ambiguity in the formation of empathic involvement. The information may add to the cognitive load since a reader must constantly update their knowledge of the reasons of why actions and emotional states of characters happen. At the same time, dialogic and interactive reading techniques have demonstrated that empathy is not an inherent feature of the text but is formed through the interaction of the reader and the text and is mediated by the contextual and developmental factors (Rogoski et al., 2025). This view is coherent with constructivist views of reading, where empathy is considered a contextual and contingent process, as opposed to being a pre-determined result of textual exposure.

Despite these theoretical and empirical advances, several research gaps remain. First, the precise mechanisms through which literary fiction may influence empathy and perspective-taking have not been conclusively established. Existing studies often conflate different components of empathy, adopt short-term experimental designs that may not capture cumulative effects, or neglect reader variability in prior reading experience and cognitive disposition (Lenhart and Richter, 2025; Schwerin et al., 2025). Second, the relationship between immediate empathic responses during reading and long-lasting changes in social behaviour has received insufficient attention. Third, the specific narrative features that may facilitate or inhibit empathic engagement such as internal focalization, character complexity, and perspective shifts have not been systematically investigated across studies (Fernandez-Quintanilla, 2020). The present review thus assesses the relationships between literary and prosocial reading and the outcomes related to empathy and social cognition, and analyzes whether the strength and direction of those relationships change as a function of the reader’s type of exposure, outcome domain, study design, and reader-engagement characteristics. However, the processes by which such linkages can be formed are presented as potential interpretations of the data in the present systems of meta-analysis, but not as causal processes directly identified through the present meta-analysis.

To address the distinct mechanisms through which different types of reading may influence empathy-related outcomes, the present review distinguishes between two primary exposure categories: literary fiction and prosocial reading. Literary fiction is distinguished by its psychological depth, character complexity, ambiguity, unreliability of narration, inferential demand and aesthetic sophistication (Kidd and Castano, 2013). Such stories tend to be morally complex, have psychologically complex characters, whose inner lives are partially revealed, and are open to interpretation, encouraging readers to make the meaning themselves (Fernandez-Quintanilla, 2020). The proposed connection between literary fiction and empathy is a process of mental effort: readers must make inferences and predictions about characters’ emotions, intentions, and beliefs based on limited information, engaging their theory of mind abilities (Kidd and Castano, 2013).

Prosocial reading refers to any text that is specifically written to demonstrate a moral behaviour, altruism, empathy or social responsibility. These are children’s literature that teaches moral lessons, health education books, value-oriented books, and books that introduce prosocial behaviours as desirable and natural behaviours (Li et al., 2022). Prosocial reading is thought to function mainly through social learning, identification with prosocial models and internalization of moral or social values, rather than the inferential demands that are specifically related to psychologically complex literary narratives (Thompson et al., 2022). A few studies focus on stories that include both literary and prosocial components (e.g., literary fiction that incorporates moral themes). These are referred to as “mixed narrative exposure. The control conditions are no reading, alternative activities, popular fiction reading, or non-fiction reading.

## 2 Literature review

Literary fiction and empathy have been widely studied in the fields of psychology, cognitive science, and literary studies, and there is a recent development of evidence that narrative engagement may have an impact on social-cognitive processes. The pioneering research by Bal and Veltkamp (2013) paper on fiction and empathy revealed that reading fictional stories can enhance empathic reactions especially when the reader has high emotions of being transported into the story (Bal and Veltkamp, 2013). This is consistent with the theory of transportation that assumes that immersion in narrative worlds can help in temporary changes of attitudes and affective states. Likewise, previous correlational research had indicated that the frequent reading of fiction correlates with better social skills and empathic sensitivity, but with weak correlations that are context-specific (Hartung et al., 2016; Ding and Lu, 2016). All these findings lead to the notion that literary fiction can play the role of a simulated social space in which readers practice interpersonal knowledge.

One of the key themes of the literature is the idea of empathy as a multidimensional construct, which is usually subdivided into affective and cognitive aspects. Cognitive empathy (usually defined as perspective-taking) is the ability to deduce the mental conditions of others, whereas affective empathy is the ability to experience the emotions of others. Neuroscientific and developmental models underline that these components are based on somewhat different mechanisms but are linked to each other in the dynamics of social cognition (Kucirkova, 2019). The dual-process model can be applied in a literary context as it is seen in the concept of narrative empathy, in which the reader creates mental images of characters, but retains a sense of self versus other. Narrative empathy, as discussed by Fernandez-Quintanilla et al. (2020) and grounded in the theoretical tradition established by Keen (2006) involves both imaginative simulation and inferential reasoning, thereby integrating concepts from cognitive narratology and social neuroscience (Fernandez-Quintanilla, 2020; Keen, 2006). Such a combined approach has enhanced the theoretical foundations of literary fiction as a field of research into intricate empathic procedures.

Empirical studies have also investigated the process by which perspective-taking happens in the process of reading. Experimental research has shown that introducing a person to a perspective of a character can led to an increase in empathic concern, prosocial behaviour, and emotional conformity, but these effects are not equally strong across individuals and contexts (Beussink et al., 2017). Stylistic devices like internal focalization, unreliable narration, and shifting perspective are said to exert greater demands in perspective-taking, making readers engage in actively reconstituting the intentions and beliefs of characters in literary fiction. This has been connected to theory of mind (TOM), a mental process that helps in the comprehension of the mental state of others. Literary fiction, as research claims, is more prone to disrupt the stereotypical thinking process, compared to popular or genre fiction and demands readers to use flexible interpretative strategies, thereby activating ToM-related processes (Santi et al., 2025).

Theoretical progress has also helped to expand the boundaries of knowledge about the role of narrative features in empathic engagement. The psychological complexity, emotional ambiguity, and multidimensional characterization commonly found in literary fiction, which present significant challenges to readers’ inferential and mentalizing capacities. This is in line with simulation theory where the readers model the experience of others internally, and constructivist descriptions that view the meaning-making process as an interactive process between the text and the reader (Rezende and Shigaeff, 2023). Multi-dimensional empirical studies which consider empathy, both affective sharing and perspective-taking, have indicated that these two dimensions can respond differently to exposure to stories. This distinction is essential because much previous research focused narrowly on cognitive empathy without adequately considering the broader emotional and ethical dimensions of empathic engagement (Huang et al., 2025; Pino and Mazza, 2016).

More current research has started to reconsider the conceptualization and measurement of empathy itself, pointing to long-standing concerns of construct validity and theoretical clarity. Researchers observe that empathy is usually inconsistently operationalized in other studies, resulting in disjointed results and comparative lack (Richaud et al., 2017). There is growing recognition that empathy should be conceptualized as a multidimensional construct embedded within broader socio-cognitive systems, rather than treated as a unitary outcome variable. It has a bearing on literary studies, in which the intricacy of narrative involvement might not be well reflected in standardized psychological examinations. As a result, the literature supporting interdisciplinary approach that incorporates the efforts of psychology, neuroscience, and literary theory to enhance the understanding of how and when literary fiction results in the formation of empathy is growing (Liao et al., 2025; Richaud et al., 2017; Surma-aho and Hölttä-Otto, 2022).

## 3 Conceptual framework and study hypothesis

The conceptual framework for the present systematic review and meta-analysis draws on theory from literary studies, cognitive psychology and social neuroscience. These hypotheses are based on a conceptual model that separates literary-fiction and prosocial-reading exposures, but permits overlap. Literary fiction is suggested to involve cognitive and inferential reading mechanisms, while prosocial reading is postulated to be more direct via identification with prosocial models and internalization of social or moral values. The proposed exposure types are hypothesized to have both cognitive, affective, and prosocial consequences, although the relative contributions of the types is tested empirically and not assumed.

### 3.1 Research rationale

It is hypothesized that literary fiction can affect social cognition in several intertwined ways. First, narratives are filled with psychologically complex characters, with ambiguous motivations and inconsistent behaviour, that require the readers to make and update their hypotheses about what the characters are thinking, feeling and doing, which involves theory of mind abilities (Kidd and Castano, 2013). Second, the experience of being transported into a story world by the narrative, where the reader experiences less self-absorption and more engagement with characters, could help to foster empathic engagement. Third, very subtle improvements in social-cognitive skills after repeated exposure to complex narratives over time can be cumulative and incremental.

### 3.2 Hypothesis

Based on the theoretical framework outlined above, the following hypotheses guide the present review:

*H1*: Exposure to literary fiction is positively associated with cognitive empathy and theory of mind performance.

*H2*: Literary fiction demonstrates stronger associations with cognitive empathy and perspective-taking than with affective empathy and prosocial behaviour.

*H3*: Narrative engagement (transportation, identification, immersion) moderates observed empathy outcomes, with stronger effects among highly engaged readers.

*H4*: Study characteristics (design type, text category, outcome measure) contribute to heterogeneity in observed effects, with pre-registered, high-powered studies showing smaller effects than earlier exploratory studies.

Empathy-related and social-cognitive effects will differ depending on reading exposure type, with literary fiction and prosocial reading being theoretically different exposure types, and potentially having different effects.

Exploratory subgroup analysis is conducted to evaluate the exposure-type hypothesis because few studies are eligible for inclusion in the prosocial-reading subgroup. The hypothesis is not designed to imply that the two exposure categories will have different effects, but that they will have measurable patterns that are different. The conceptual model underlying these hypotheses is presented in Figure 1. This model proposes that the layered narrative structure of literary fiction requires cognitive effort that in turn facilitates Theory of Mind (ToM) and cognitive empathy. Affective empathy and prosocial behaviours are hypothesized as secondary effects dependent on particular reader-state variables (e.g., narrative transportation and character identification) and contextual factors.

**Figure 1:**
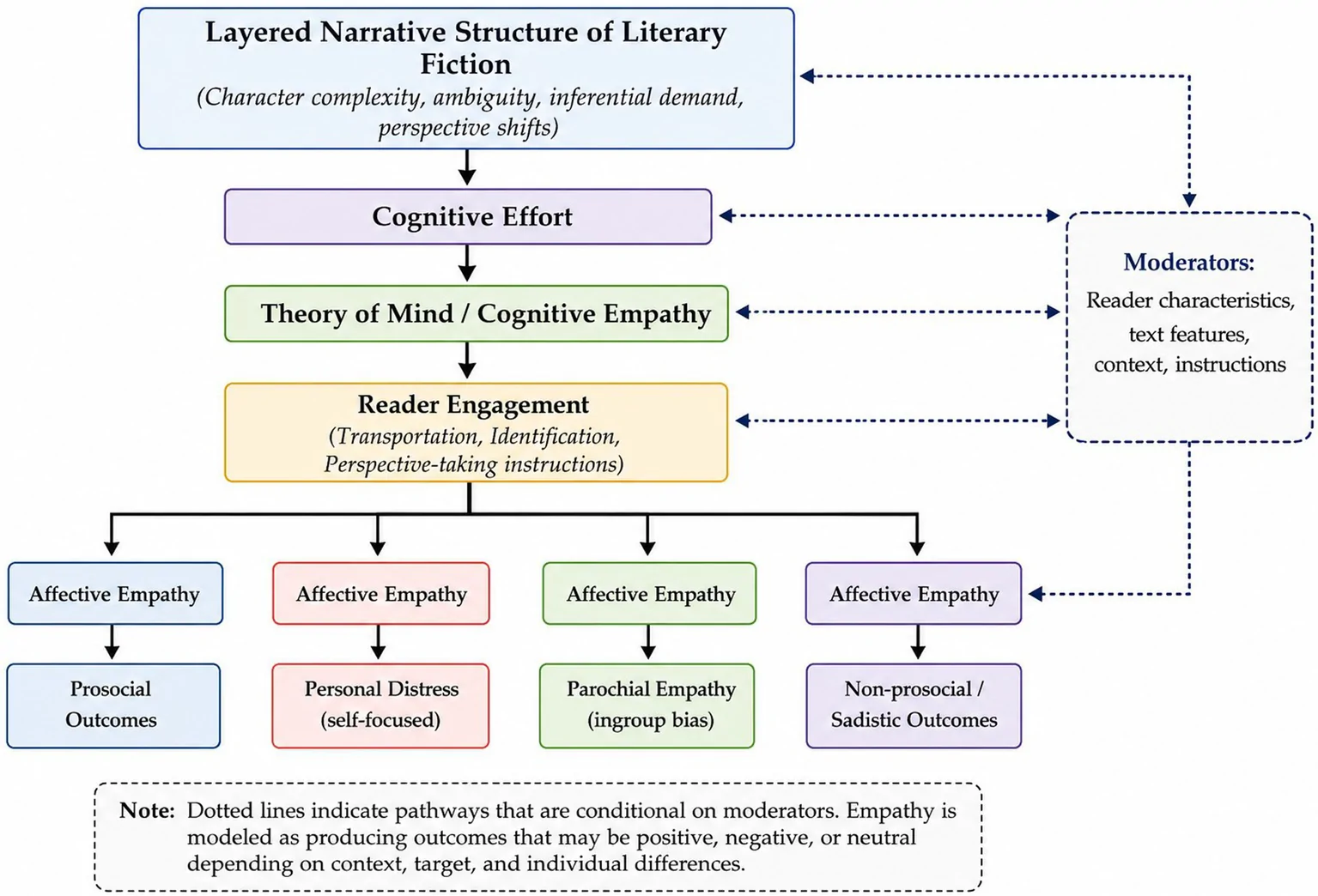
Conceptual model of cognitive and affective pathways in literary reading with context-dependent outcomes. Path diagram illustrating the hypothesized mechanisms by which literary fiction influences cognitive empathy and theory of mind via narrative structure and cognitive effort. Affective empathy and prosocial outcomes are modeled as secondary, reader-dependent processes moderated by transportation, identification, and individual differences. Affective empathy may produce positive (prosocial), negative (personal distress, non-prosocial), or parochial (ingroup bias) outcomes depending on context, target, and individual differences. Dotted lines indicate pathways conditional on moderators. Adapted from theoretical frameworks in literary studies, cognitive psychology, and social neuroscience.

## 4 Methods

### 4.1 Research design

The study is a systematic review with an embedded quantitative meta-analysis to clarify how literary and prosocial reading relate to empathy and related social-cognitive outcomes. The review collates work on narrative reading that reports quantitative data on constructs such as empathy, perspective-taking, theory of mind, and prosocial behaviour. Experimental, quasi-experimental, correlational, and neurocognitive designs are all included, provided they meet clearly defined criteria. The goal is not only to estimate average effects, but also to describe how those effects vary across designs and conditions. The review process follows contemporary standards for transparent and reproducible evidence synthesis, including explicit documentation of search, screening, and analytic decisions.

### 4.2 Protocol and preregistration

This review was not a prospectively registered review. But eligibility criteria, study selection procedures, outcome classification categories and data extraction protocols were defined prior to the formal data extraction process. The conceptual model/hypotheses outlined in the Introduction were *a priori* developed based on the theoretical literature, although inductive insights from the included studies were also taken into account in the synthesis.

### 4.3 Search strategy

The literature search involved five large databases that are popular in the field of psychology, education, and other areas: PubMed, Scopus, Web of Science, PsycINFO and ERIC (Supplementary Figure S1). It was between January 2000 and early 2025, which covered both early foundational research and the latest work on pre-registering and neurocognitive research. Search strings were word combinations of terms that described narrative exposure (such as, but not limited to, literary fiction, narrative reading, fiction exposure, perspective-taking, theory of mind, mindreading, prosocial behaviour, helping, moral identity) and Boolean operators. Database filters were used where possible to limit results to human subjects and peer-reviewed journal articles.

Additional measures were taken to minimize the chances of overlooking pertinent work. Included articles and related reviews had reference lists which were screened to identify further studies. Influential papers were tracked forward with citation to identify subsequent work based on them. Where dissertations or reports were found in searches, where possible, peer-reviewed journal versions of the same data were sought.

### 4.4 Eligibility criteria

Prior eligibility criteria were set. The inclusion criteria were as follows: The studies included had to fulfill all the following criteria: Reported empirical quantitative data, either single or as part of a mixed-method design, Published in peer-reviewed journals. Had some literary, narrative or explicitly prosocial reading element of central exposure or predictor, manipulated experimentally (e.g., literary fiction vs. nonfiction) or measured as a habit (e.g., fiction print exposure, prosocial reading frequency). Evaluated at least one outcome of empathy (e.g., cognitive empathy, perspective-taking, theory of mind), affective empathy (e.g., empathic concern, state empathy), or prosocial outcome (e.g., helping behaviour, prosocial intentions, moral identity). Adequate statistical data to compute or estimate effect sizes (e.g., means, standard deviations, sizes of groups, *t* or *F* statistics, odds ratios, or zero-order correlations).

Articles were filtered out due to the fact that they either only involved non-reading media (e.g., film or games), only measured non-social outcomes (e.g., decoding skills or vocabulary without social-cognitive measures) or did not provide extractable statistics. In cases where several papers made use of the same sample, the most comprehensive and methodologically clear report was left to be used in effect-size extraction, with other reports being summarized with only secondary information.

### 4.5 Screening and study selection

The screening was done in two phases. The first step was to filter titles and abstracts and eliminate clearly ineligible records, like those that studied technical reading skills without social outcomes, non-human studies or entirely theoretical contributions. At this point, the reference of fiction or narrative reading along with social, emotional, or moral construction stimulated retention in the full-text evaluation. During the second phase, full texts were reviewed in accordance with the eligibility criteria, and the causes of exclusion were recorded (e.g., incorrect outcome, non-narrative intervention, lack of statistics). The result of this process was a PRISMA-style flow of records between identification and inclusion. The final eligible sample is consisted of a total of 21 primary studies that were incorporated in the qualitative synthesis, of which 12 studies (14 effect sizes) had enough data to be incorporated in the quantitative meta-analysis.

### 4.6 Data extraction and coding

Data extraction was done under a structured protocol that would extract similar data in each study. Extracted variables included:

Bibliographic information: names of authors, the year of publication, the journal, and the country or region.
Characteristics of the sample: overall sample size and, where possible, age span, educational attainment and gender distribution.
Design of the study: randomized experiment, quasi-experiment, intervention study, correlational survey, neuroimaging study, or reader-response study with quantitative results.
Reading exposure: type of text (e.g., literary short story, novel passage, no reading), control conditions (e.g., nonfiction, popular fiction, no reading), and, in correlational studies, the operationalization of reading (e.g., author recognition test, self-reported fiction reading).
Outcome measures: specific empathy measures, perspective-taking or theory-of-mind measures, affective empathy measures, and prosocial outcomes; names of scales and type of task were noted.
Statistical data: means, SDs, group sizes, *t*-statistics, *F*-statistics, zero-order correlations, regression coefficients, and other data necessary to compute the effect-size.

In cases where more than one outcome of interest was reported in a particular study, each was given a unique coding as an outcome but attached through a shared study identifier. Dependency in meta-analytic models was addressed with selection or aggregation strategies that would be suitable considering the construct being considered.

### 4.7 Outcome direction and contextual coding

The direction of the outcome (beneficial vs. harmful) was determined separately from the type of evidence (empathy related vs. non-empathy related). The original research interpreted the empathy, perspective taking, theory of mind or prosocial behaviour increase as beneficial or other-oriented and the research was classified as prosocial/beneficial. Nonsignificant, negligible, or inconsistent findings were coded as null/weak. Findings were classified as non-prosocial or context-dependent when increased empathic responding was correlated with self-oriented distress, ingroup preference, intergroup bias, counter-empathic responses, or other results that were not generally prosocial. The mechanistic evidence was kept as a separate evidence-type descriptor because mechanistic evidence does not necessarily point to a behavioural direction. Allowing these coding dimensions to overlap was done because a study might have presented and reported both a positive and a null or context-dependent behavioural outcome, as well as a neurocognitive outcome.

### 4.8 Assessment of evidence grading

Each hypothesized pathway (Table 1; Figure 2) was evaluated for the strength of evidence, based on a set of predefined qualitative criteria, which draw on multiple dimensions of the available evidence. The evidence strength framework used in this review is a study specific qualitative synthesis rubric and is not an indication of a formal assessment of evidence by the GRADE framework. It was created because the review incorporates studies with heterogeneous types of evidence (randomized studies, quasi-experimental, correlational, and neurocognitive) and because the hypothesized pathways cannot be easily translated to a traditional intervention-certainty model. The rubric reflects known principles of evidence-assessment, such as the need to consider consistency, methodological quality, replication, precision, and convergence across the study designs but should not be understood as equivalent to formal GRADE certainty ratings. The criteria were developed before evidence is classified, to maintain transparency and reproducibility.

**Table 1:** Hypothesis mapping table.

Hypothesis component	Supporting evidence	Strength
Literary fiction enhances cognitive empathy/ToM	Kidd and Castano (2013), Mar et al. (2006, 2009), Pino and Mazza (2016), Fekete et al. (2023)	Strong (with some qualified by later null findings)
Affective empathy depends on engagement	Bal and Veltkamp (2013), Green and Brock (2000), Sestir and Green (2010)	Strong
Effects are not universal	Samur et al. (2018), Lenhart and Richter (2025), Kidd and Castano (2019)	Strong
Neural mechanisms underpin empathy/ToM in fiction	Tamir et al. (2016), Nijhof and Willems (2015), Vaughn et al. (2018)	Moderate–Strong
Long-term exposure more effective than single sessions	Mar et al. (2006, 2009), correlational evidence on print exposure	Moderate

Evidence strength ratings are based on predetermined criteria, as outlined in Methods (Assessment of Evidence Strength). HIGH = many good quality studies, similar results when using different methods, and a statistically sound meta-analysis. MODERATE = vague or inconsistent support, and of limited replication. LOW = low levels of support from empirical findings, inconclusive findings, or lack of quality empirical evidence.

**Figure 2:**
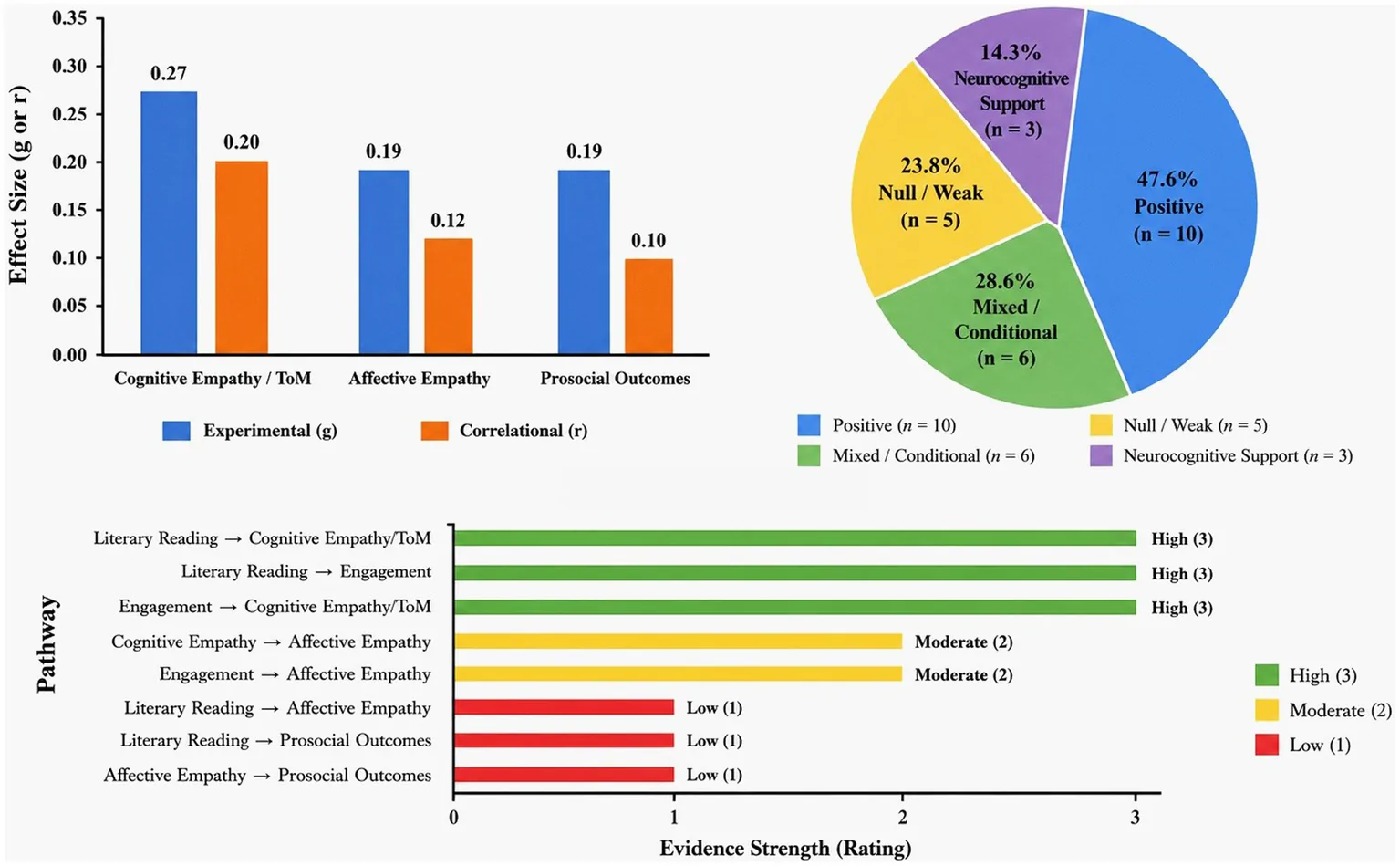
Summary of effect sizes, outcome distribution, and evidence strength. **(A)** Approximate effect sizes (standardized) for experimental (Hedges’ *g*) and correlational (Pearson’s *r*) studies across key outcomes. Values are based on the corrected meta-analysis (*k* = 14 effect sizes from 12 studies). Cognitive empathy and theory of mind show the largest effects (*g* = 0.27), while affective empathy (*g* = 0.19) and prosocial outcomes (*g* = 0.19) show smaller but still positive effects. **(B)** Distribution of studies by outcome type across the 21 primary studies included in the systematic review (Table 2). Positive effects were reported in 47.6% of studies (*n* = 10), mixed or conditional effects in 28.6% (*n* = 6), null effects in 23.8% (*n* = 5), and neurocognitive support in 14.3% (*n* = 3). Percentages sum to >100% because some studies are counted in multiple categories. **(C)** Evidence strength ratings for hypothesized pathways in the conceptual model. Evidence strength was rated as High (3), Moderate (2), or Low (1) based on the consistency and robustness of available empirical support, as outlined in the Methods section. The strongest evidence supports the pathway from literary reading to cognitive empathy and theory of mind (*g* = 0.27, *p* = 0.002), while evidence for direct pathways to affective empathy (*g* = 0.19, *p* = 0.004) and prosocial outcomes is weaker and more context-dependent.

HIGH (Strong) evidence:

- Consistent support from at least three independent studies rated as having low or moderate risk of bias according to the study-design-specific assessment.
- Convergent evidence - across multiple methodological approaches (experimental, correlational, and as available, neurocognitive).
- On the other hand, statistically reliable meta-analytic results with confidence intervals that do not intersect with 0 are included.
- The consistency of directions and magnitudes of effects among independent research groups.
- Frequent replication in pre-registered studies. Reliable replication of pre-registered studies.

MODERATE evidence:

- Mostly positive results with some variability among studies.
- Significant methodological differences hindering cross-comparison.
- Limited replication, with support from only one or two studies rated as having low or moderate risk of bias.
- Evidence mostly based on one method of analysis.
- Meta-analytic results that show some uncertainty (confidence intervals are not overlapping, but are wide).

LOW evidence:

- Limited empirical support.
- Differences in results among studies.
- Lack of well-documented evidence to make strong conclusions.
- The results of meta-analyses that include confidence intervals that cross the value of zero or on studies with few effect sizes.
- Evidence from exploratory or inadequate studies is the primary basis for the judgments.

Application of Criteria Each hypothesized pathway was evaluated according to these criteria, based on the evidence and the quantitative meta-analytic results from Section 4.9 to 4.12. The level of evidence for each pathway was classified by two independent assessors (the first author and a research assistant) with disagreements resolved by discussion. This structured synthesis process is reflected in the ratings given in Table 1 and Figure 2. What is important to note is that these criteria are qualitative, and not imposed arbitrarily as a percentage of significant studies or as a minimum size of effect criterion. The methodological variations among the studies in the literature and the limited number of studies in some evidence categories would not allow for a single number to reliably capture the quality of evidence and might misrepresent the empirical uncertainty. The qualitative criteria, using a clear and explicit dimension and definition, offers a clear, transparent, and replicable evidence assessment framework. Accordingly, the ratings summarize the strength of support for a hypothesized pathway rather than the formal certainty of an intervention effect.

### 4.9 Exposure classification

The reading exposures were sorted into one of four categories according to the type of text in each major study:

Literary fiction (LF): Studies based on excerpts from literary short stories, novel passages, or a work of literary fiction that are selected for their psychological depth, character complexity, and interpretive openness.
Prosocial reading (PR): Research conducted on narratives that have been intentionally created to represent prosocial behaviour, ethical principles or empathy (such as moral stories for children, health education stories).
Mixed narrative exposure (MIX): Research studies with narratives that include both literary and prosocial elements.
Non-reading/control (CTRL): Studies which use non-fiction reading, popular fiction, no reading and/or alternative activities as comparison conditions.

It is important to note that the exposure is not the comparison exposure, but rather the primary reading exposure. Therefore, a study, comparing literary fiction with nonfiction, was coded as literary-fiction condition and the nonfiction condition was coded as a control condition. Classification has been determined according to the text description presented in each of the primary studies or on the theoretical basis of the selection of text. Two independent coders (the first author and a research assistant) classified each study; disagreements were resolved through discussion.

### 4.10 Effect size computation

Reported statistics were transformed into common effect-size measures to facilitate synthesis across a variety of designs and measures. In group-comparison designs (e.g., literary fiction vs. control) the standardized mean differences were computed and corrected against small-sample bias with the Hedges *g*. Where *t* or *F* statistics were only available, equivalent standardized differences were obtained using standard formulas. In the case of correlational designs, Pearson *r* has been extracted or derived and was converted to *z* of Fisher to do meta-analytic calculations, because it has a more desirable sampling distribution. The orientations of effect sizes were always in the positive direction in which higher empathy, improved perspective-taking or theory-of-mind performance, or more prosocial results were observed to be related to literary or prosocial reading. Upon analysis, it was interpreted that pooled effects were in both *g* and *r* measures.

### 4.11 Statistical analysis

Random-effects models were used to conduct quantitative analyses, and it was assumed that true effect sizes might differ among studies as a result of differences in design, populations, texts, and measures. The effects sizes were weighted by the inverses of variance with more accurate estimates carrying more weight. The main model lumped together all the pertinent outcomes related to empathy. Secondary models considered smaller subsets, e.g., cognitive empathy and theory-of-mind outcomes or affective and prosocial outcomes.

The heterogeneity between studies was assessed with *Q* statistic and *I*^2^ index. *Q* was used to test whether the dispersion observed was greater than would be due to sampling error alone, and *I*^2^ measured the percentage of total variance due to real differences between studies, and not due to chance. Forest plots were created to show individual and pooled effect sizes with 95 percent confidence interval. In the case where the number of studies allowed, exploratory subgroup analyses and basic meta-regressions were carried out to assess whether a design type, text category, or an engagement-related variable was a cause of heterogeneity.

Subgroup analyses were considered exploratory as exposure type and outcome domain are study level characteristics that are not necessarily independent. In particular, there may be some overlap in the comparison of outcome subgroups and exposure subgroups due to the composition of the available studies. So, the estimates for the subgroups are not used as tests of mutually exclusive causal mechanisms but simply as descriptions of the available evidence. Between-subgroup heterogeneity was evaluated using Q_between, and differences were considered statistically meaningful only when the formal between-subgroup test was significant.

### 4.12 Evaluation of publication bias and study quality

All potential publication bias and small-study effects were explored visually and statistically. Effect sizes were plotted as funnel plots versus the standard errors and tested against asymmetry with the regression test of Egger and the rank correlation test of Begg. These tests were viewed with reservations considering the small number of studies. The quality of studies and the risk of bias were evaluated independently of the types of designs. In randomized and quasi-experimental studies, selection bias (e.g., randomization and allocation procedures), performance and detection bias (e.g., awareness of condition, blinding of assessor where relevant), attrition and selective reporting were important domains. In correlation studies, the focus was on sampling procedures, reliability measures of the measurements, and adjustment of the obvious confounders. Neuroimaging articles were rated based on sample size, articulateness of analysis pipelines, and multiple comparisons correction. The judgments of risk-of-bias were summarized by category, as opposed to being reduced to a single composite score.

## 5 Results

### 5.1 Characteristics of included studies

The final 21 studies cover more than 20 years, and represent an evolution in research on fiction and empathy (Figure 3). The initial studies tended to use correlational designs, linking measures of exposure to fiction - such as author name recognition tests or self-report measures of reading habits to empathy measures and theory-of-mind tasks. As interest grew, more experimental studies began to appear, often comparing the effects of literary short stories or passages from novels to non-fiction or popular-fiction literature on social-cognitive performance in the immediate aftermath. Recent have seen an increase in highly powered pre-registered experiments and studies using neuroimaging to understand the role narratives play in the brain’s social networks (Table 2).

**Figure 3:**
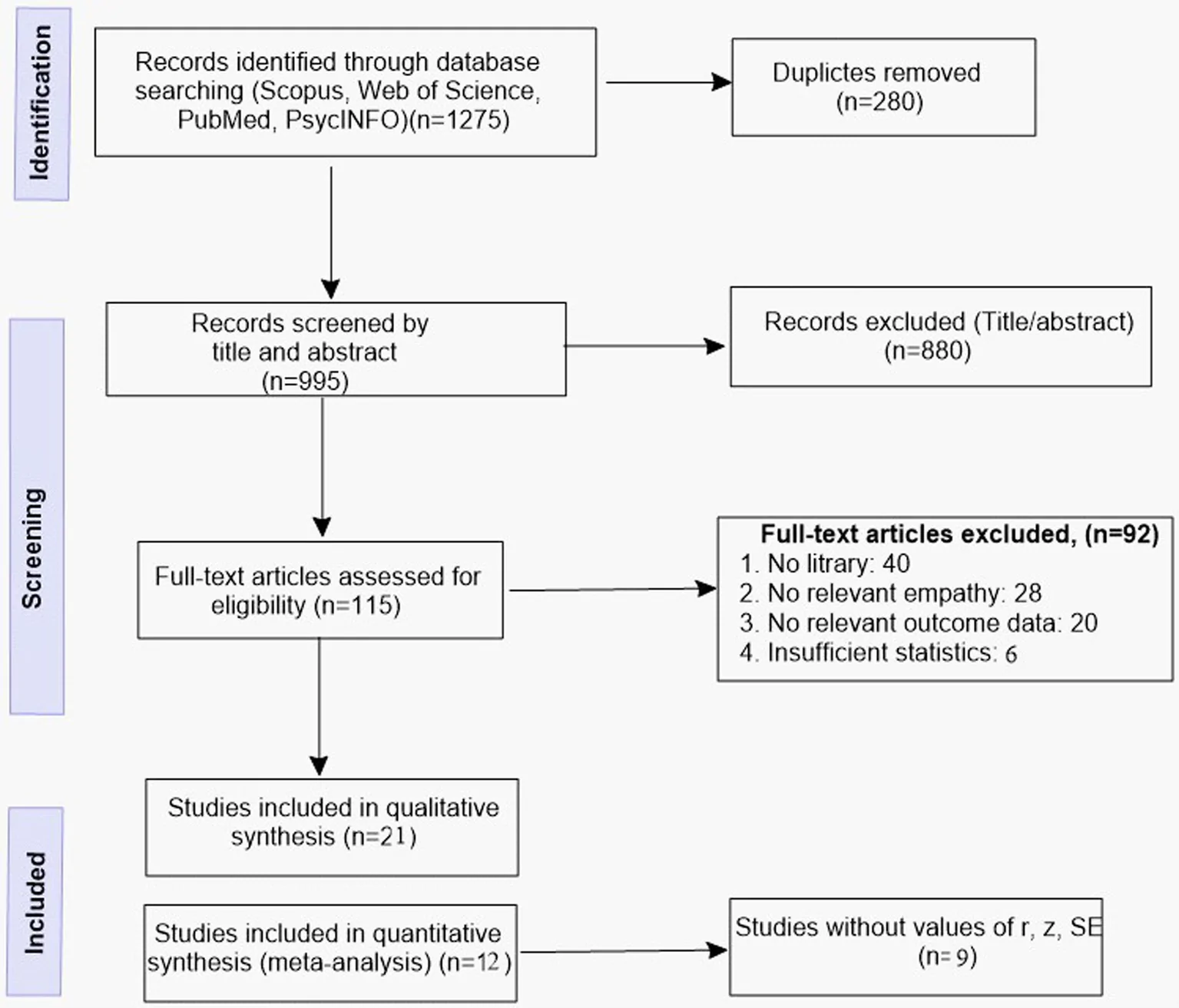
PRISMA flow diagram of study selection. Flow chart showing the number of records identified, screened, excluded (with reasons), and included in the qualitative synthesis and quantitative meta-analysis, following PRISMA 2020 guidelines.

**Table 2:** Primary studies on empathy, perspective-taking, and prosocial outcomes in literary/prosocial reading.

Study	Country	*N*	Design	Measures	Key findings	Outcome	Exposure type
Bal and Veltkamp (2013)	Netherlands	66, 97	Exp. Longitudinal	Empathy, transportation	Empathy ↑ only under high immersion	Conditional (affective)	Mixed
Beussink et al. (2017)	United States	296	Experimental	Empathy, sadness, helping	“Imagine-self” ↑ empathy, esp. high CA	Conditional (PT)	Literary fiction
Fernandez-Quintanilla (2020)	Spain	248	Qualitative	Self-reported empathy	Text × reader context shapes empathy	Context-dependent	N/A (qualitative)
Fekete et al. (2023)	Hungary	47/48	Experimental	ToM tasks	Story intervention improved ToM	Positive (cognitive)	Literary fiction
Green and Brock (2000)	United States	97/69/274/258	Experimental	Transportation	Immersion predicts empathy/beliefs	Mediated (transport)	Literary fiction
Kidd and Castano (2013)	United States	86/114/69/72/356	RCT	RMET, ToM	Literary fiction ↑ ToM performance	Positive (cognitive)	Literary fiction
Kidd and Castano (2019)	United States	478/381/432	Replication	ToM tasks	Small, inconsistent effects	Weak/null	Literary fiction
Koopman (2015)	Netherlands	210	Experimental	Empathy scales	Reader traits moderate outcomes	Conditional	Literary fiction
Kuzmičová et al. (2018)	Europe	36/21	Qualitative	Reading context	Social context shapes experience	Contextual	N/A (qualitative)
Lenhart and Richter (2025)	Germany	224	Experimental	Empathy, ToM	No differences across conditions	Null	Literary fiction
Li et al. (2022)	China	602	Survey	Prosocial behaviour	Reading → PB via moral identity	Mediated positive	Prosocial reading
Liao et al. (2025)	United States	219, 372	Experiments	State empathy	Distress → empathy → attitudes	Mediated positive	Literary fiction
Mar et al. (2006)	Canada	94	Correlational	ToM, empathy	Fiction exposure ↑ ToM/empathy	Positive association	Literary fiction
Mar et al. (2009)	Canada	252	Correlational	Empathy scales	Effect persists after controls	Positive association	Literary fiction
Nijhof and Willems (2015)	Netherlands	18	fMRI	ToM networks	Distinct neural reading styles	Neurocognitive	Literary fiction
Pino and Mazza (2016)	Italy	214	Experimental	RMET, empathy	Literary fiction ↑ mentalizing	Positive (cognitive)	Literary fiction
Rogoski et al., 2025	Brazil	12	Intervention	Empathy scales	Intervention ↑ empathy	Positive	Prosocial reading
Samur et al. (2018)	Netherlands	160	Replication	RMET	No reliable ToM improvement	Null/weak	Literary fiction
Sestir and Green (2010)	United States	118	Experimental	Transportation, identity	Identification shifts self-concept	Conditional	Literary fiction
Tamir et al. (2016)	United States	26	fMRI	Default network	Fiction engages ToM networks	Neurocognitive	Literary fiction
Vaughn et al. (2018)	United States	135	fMRI	Empathy networks	Ingroup bias in empathy	Neurocognitive	Literary fiction

Participants ranged from small-scale, intensive studies (a dozen or so individuals) to large-scale surveys (hundreds of participants). Large surveys with hundreds of participants. Most studies involved adolescent or studies have focused on adolescents or adults, most commonly university students, but also research looked at children or more representative community samples (Table 2). Geographically, most studies were conducted in North America and Europe, with some studies from South America and East Asia. Across different cultures and methods, all studies had a central concern with the ways reading narratives can be connected with understanding, feeling for or acting toward others.

### 5.2 Study characteristics

The empirical evidence testing the pathways depicted in this model is outlined in the overview of primary studies (Figure 4), a visual supplement to the study characteristics outlined in Table 2. There is a strong cluster of studies that report positive or mediated effects (green and orange dots, respectively), especially in experiments. Although this field is dominated by experiments, the addition of correlational and neuroimaging studies allows a multimodal view of literary engagement. It is important to note that the weak or absent effects (red markers) are connected to the recent high-powered or pre-registered replication, a pattern suggesting the need for more sophisticated moderator-focused analyses, such as those in the next sections of this report.

**Figure 4:**
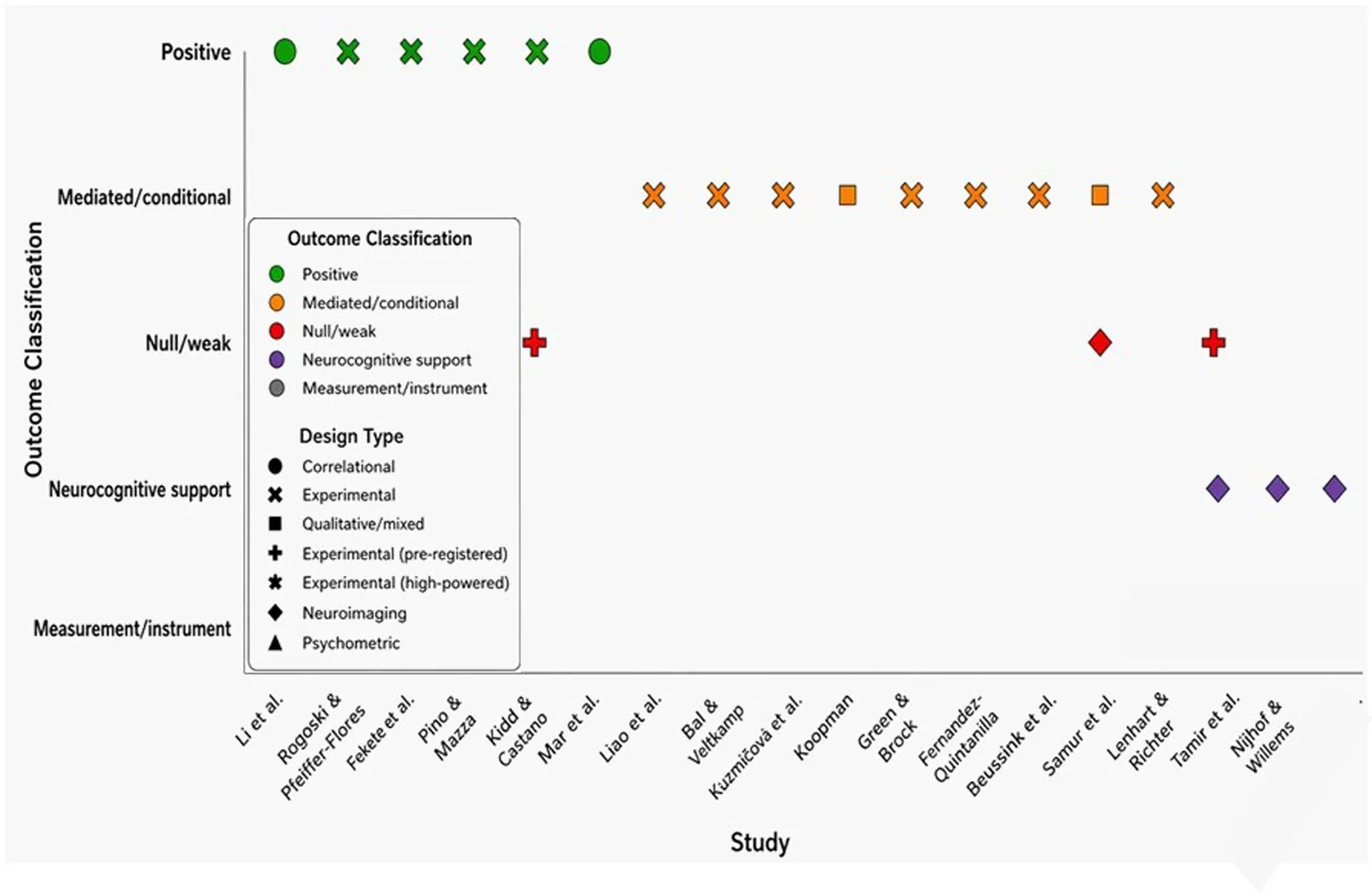
Overview of primary studies on empathy, perspective-taking, and prosocial outcomes. Graphical summary displaying each study along the x-axis and classifying them by outcome type (positive, mediated/conditional, null/weak, neurocognitive support, measurement/instrument) using color-coding or grouped bars, with icons or shading to distinguish design types (experimental, correlational, neuroimaging, qualitative).

### 5.3 Descriptive patterns of outcomes

Categorising studies according to headline findings is illuminating. A substantial fraction found definitive positive effects/associations with literary/prosocial reading and empathy-related outcomes (47.6%, *n* = 10) (Table 3; Figure 5). These studies included experiments where participants who read literary fiction scored higher on theory-of-mind measures than did participants who read nonfiction, and correlational studies in which fiction reading was positively associated with empathy and social-cognitive measures. A second group of studies found conditional effects (28.6%, *n* = 6). In these situations, change did not result from text type alone; rather, it seemed to occur mainly when readers were transported into the story, were highly identified with characters, or were given followed explicit instructions to imagine themselves in a character’s place. Statistical analyses in this work tended to find significant indirect effects via transportation or identification, or interactions of reader characteristics and conditions.

**Table 3:** Statistical synthesis of outcomes.

Outcome category	No. of studies	Percentage (%)	Interpretation
Positive effect	10	47.6%	Supports hypothesis that reading/prosocial content can enhance empathy, ToM, or prosocial outcomes.
Mediated/conditional effect	6	28.6%	Effects depend on transportation, identification, perspective-taking, or reader characteristics.
Null/weak effect	5	23.8%	No consistent short-term impact of brief exposure on empathy/ToM.
Non-prosocial/context-dependent outcome	1	4.8%	Ingroup bias, self-oriented distress, counter-empathic or otherwise non-uniformly prosocial response
Neurocognitive support	3	14.3%	Provides biological and mechanistic plausibility for fiction-related empathy/ToM processes.

Outcome direction and evidence type were coded separately and could overlap. A study could therefore contribute to more than one category, particularly when it reported both behavioural and neurocognitive findings or when effects were conditional on reader or contextual factors. Non-prosocial/context-dependent coding was used when increased empathic responding was associated with self-oriented distress, ingroup preference, intergroup bias, or other outcomes that could not be interpreted as uniformly prosocial.

**Figure 5:**
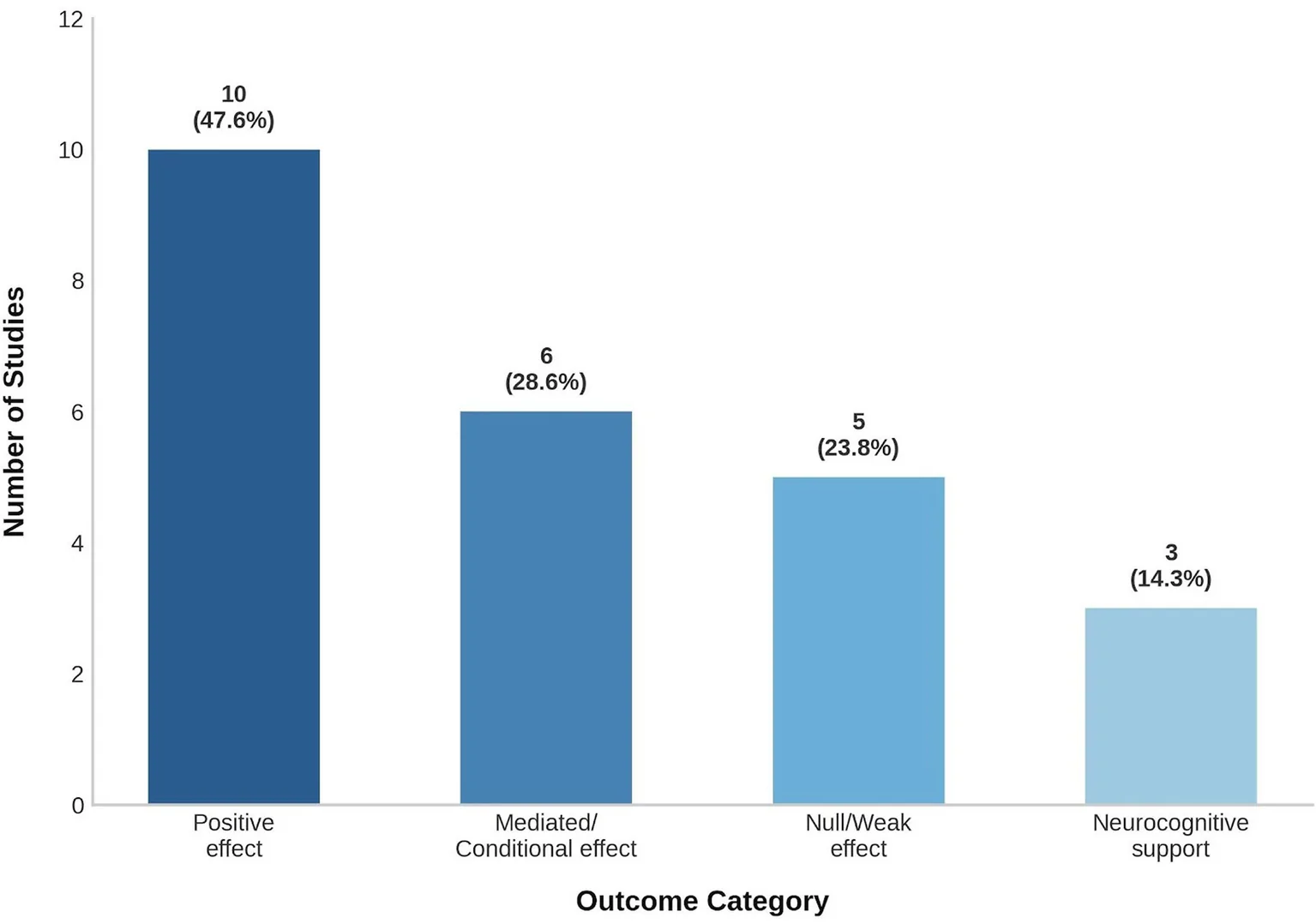
Distribution of outcome categories across primary studies. Bar chart reflecting the proportions of studies reporting positive effects, mediated/conditional effects, null/weak effects, and neurocognitive support. Nearly half of the studies report positive effects (47.6%, *n* = 10), and around a quarter show mediated or conditional patterns (28.6%, *n* = 6).

A third group, predominately among recent and pre-registered studies, found small or literary or prosocial texts to controls on standard tasks. These finding larger sample sizes, more rigorous analyses, and in some cases equivalence tests to argue that it is very unlikely that a single reading session could have a large effect on either empathy or theory-of-mind performance. Studies employing equivalence testing concluded that it is very unlikely that a single reading session could produce a large effect on empathy or theory-of-mind performance (Table 3). Finally, a handful of neurocognitive studies provided mainly mechanistic evidence, showing mentalizing and simulation network activation during narrative processing, even though behavioural effects were minimal.

### 5.4 Mechanistic and neurocognitive evidence

Convergent mechanistic support was given by neurocognitive studies. The functional neuroimaging studies demonstrated that reading literary stories involves brain areas related to mentalizing (including medial prefrontal cortex and temporo-parietal junction) and in some readers sensorimotor areas related to embodied simulation. In a single study, frequent fiction readers showed enhanced activation in default-mode networks as well as social-cognitive networks on narrative tasks and superior theory-of-mind tasks, and neural activity partly mediated the relationship between the frequency of reading and social-cognitive performance (Table 2). Other researchers differentiated between primarily mentalizing and primarily simulating readers, with divergent neural profiles in processing a narrative.

Though the behavioural shifts in the case of individual experiments tended to be relatively small, these neural results provide a plausible explanation of how the continued exposure to complex stories can eventually influence the social-cognitive operations (Table 4). Literary reading can over time lead to small, but significant changes in perspective-taking and related skills because of repeated recruitment of mentalizing and simulation systems.

**Table 4:** Risk of bias assessment.

Study category	Selection bias	Performance bias	Measurement bias	Overall risk
Randomized experiments	Low	Moderate	Moderate	Moderate
Pre-registered studies	Low	Low	Low	Low
Correlational studies	High	Moderate	High	High
Neuroimaging studies	Moderate	Low	Moderate	Moderate

Risk of bias was judged at the category level (not individually per study).

### 5.5 Risk of bias and study quality

The risk of bias differed across study types (Table 4; Figure 6). Randomized experiments had low selection bias (because of clear random allocation) but moderate performance and measurement biases were estimated due to lack of blinding and self-report measures were frequently used. Pre-registered studies were the best category, with low risk for selection, performance and measurement bias, due to tight control of analytic flexibility and reporting of results. Restrictions on analytic and outcome reporting. Correlational studies had high risk of selection and measurement bias, as studies relied on convenience samples, self-reported exposure, and cross-sectional designs that limit causal inference. Neuroimaging studies were ranked as moderate quality: used accurate measures of neural activity but often had small samples and complex statistical analyses. These patterns indicate that studies on causal contributions of literary fiction to empathy are best supported by the preregistered experimental research, but the correlational and neurocognitive results have higher implications for long-term associations and mechanisms rather than demonstrating causal impact.

**Figure 6:**
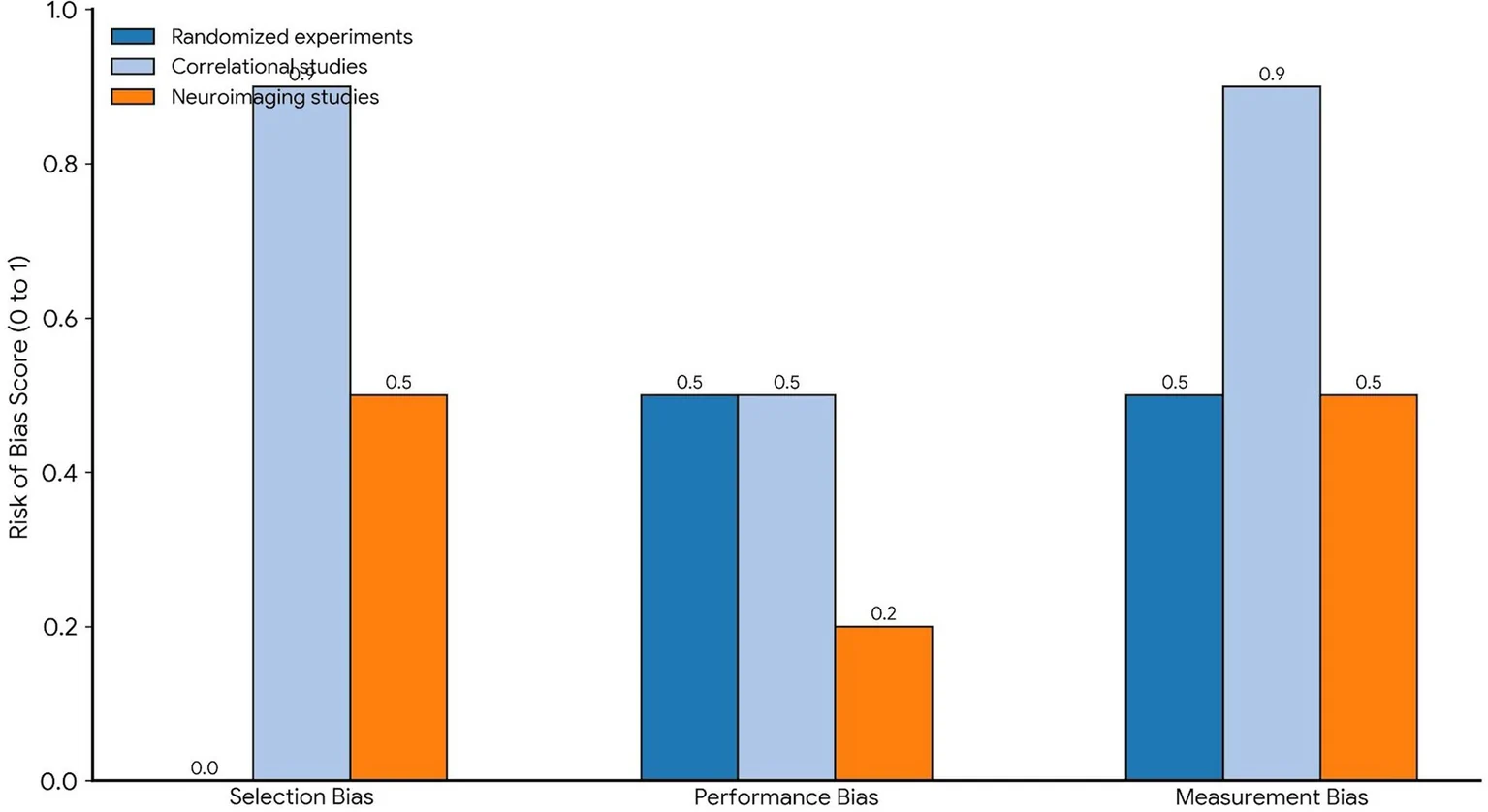
Risk of bias profile by study category. Clustered bar chart showing selection, performance, and measurement bias levels for each study category (randomized experiments, pre-registered studies, correlational studies, neuroimaging studies). Risk levels are coded as 0.0 = low, 0.5 = moderate, and 0.9 = high. Pre-registered studies show the lowest risk across all domains (0.0), while correlational studies show the highest risk for selection and measurement bias (0.9). Randomized experiments show moderate risk (0.5) for performance and measurement bias due to lack of blinding and use of self-report measures. Neuroimaging studies show moderate risk (0.5) for selection and measurement bias due to small sample sizes. Risk of bias was judged at the category level (not individually per study).

### 5.6 Temporal trends in the evidence base

Progress over time in the study of literary fiction and empathy showed a developmental trajectory (Table 5; Figure 7). The early studies (2000–2009: three studies) were largely involved initial correlational work that established a connection between fiction exposure and empathy and theory of mind. Between 2010 and 2015 (seven studies), there was a prolific growth of experimental research testing the causal effects of reading literary fiction on ToM and empathy. Since 2016 (six studies), the focus has shifted to replication, replication with a critical eye, and high-powered designs, with generally weaker or no effects compared to the earlier more positive results. The most recent years (2021–2025; five studies) have seen and focus on neural mechanisms, moderated and mediated effects, and more distinguishing cognitive and affective aspects of empathy. Overall, this trajectory points to a move away from overarching claims of large positive effects to a more sophisticated picture of smaller, conditional effects substantiated by multiple research methods.

**Table 5:** Year-wise distribution of studies.

Period	No. of studies	Percentage
2000–2009	3	14.3%
2010–2015	5	23.8%
2016–2020	8	38.1%
2021–2025	5	23.8%
Total	21	100%

**Figure 7:**
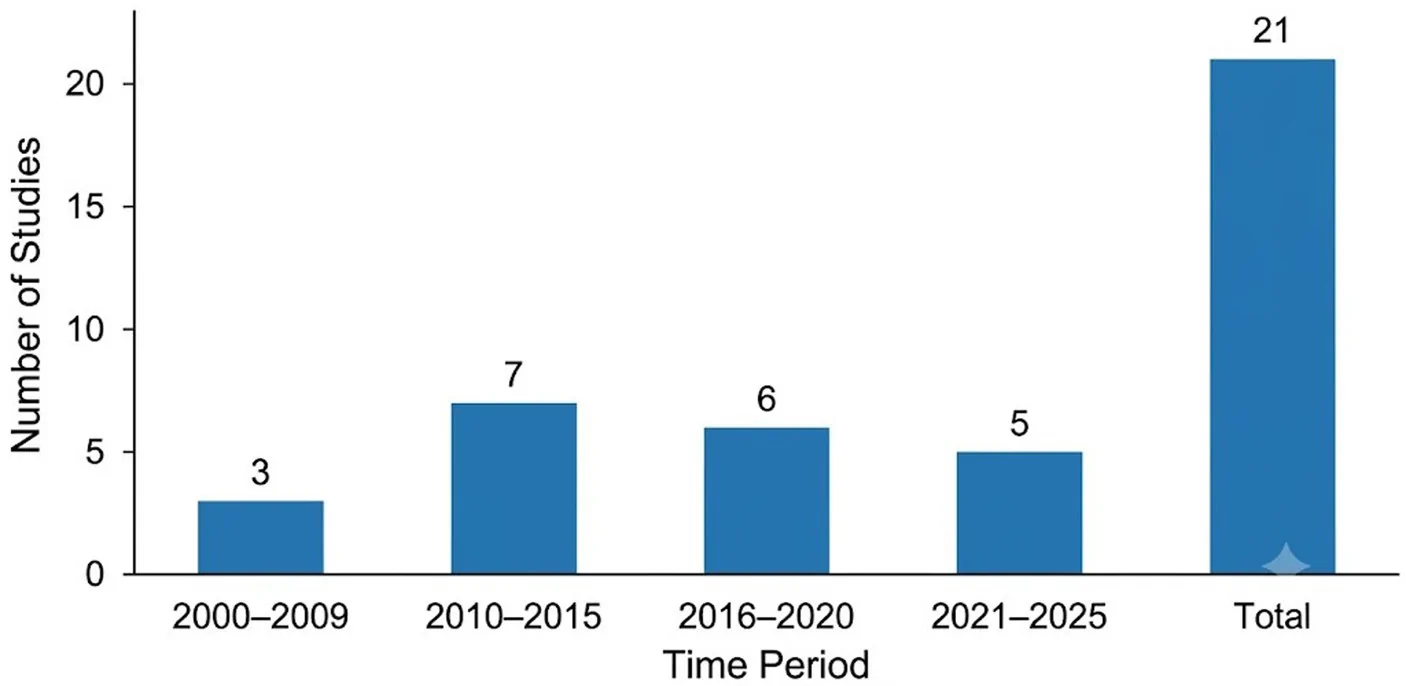
Year-wise distribution of studies on literary fiction and empathy-related outcomes. Bar chart plotting the number of studies in each period (2000–2009, *n* = 3; 2010–2015, *n* = 7; 2016–2020, *n* = 6; 2021–2025, *n* = 5), highlighting the expansion phase (2010–2015), replication and critical evaluation period (2016–2020), and recent neurocognitive and mechanistic work (2021–2025).

### 5.7 Hypothesis mapping and integrative interpretation

The links between primary studies and major elements of the theoretical model are indicated in Table 1 and represented graphically in Figure 8. Several experimental and correlational studies provide evidence that literary fiction improves cognitive empathy and ToM, although recent replication studies indicate that the average effect is modest. Evidence for the role of engagement in affective empathy points to the roles of transportation, identification, and specific perspective taking instructions. High-powered replications and equivalence tests also show that effects are not ubiquitous, and that the effects of brief exposures lacking engagement are not necessarily positive. Neuroimaging studies also indicate that fiction reading activates simulation and ToM systems, suggesting a possible mechanism for both cognitive and affective effects. Finally, correlational studies of long-term print exposure indicate that over time, reading habits may be especially important (see Figure 9; Table 6).

**Figure 8:**
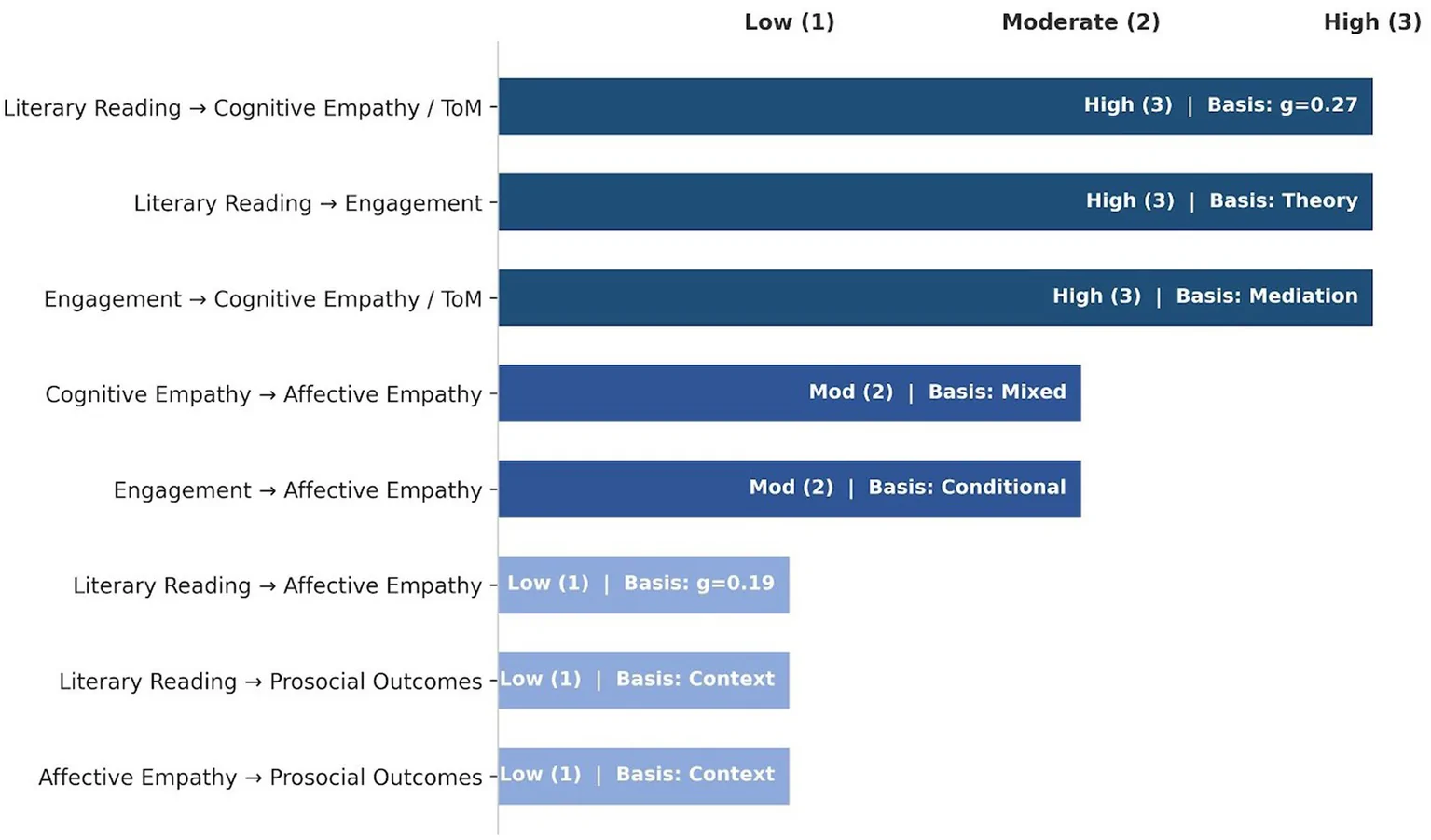
Hypothesis mapping across cognitive, affective, and neural evidence. Conceptual evidence map showing which sets of studies support each hypothesis component. Evidence strength was rated as High (3), Moderate (2), or Low (1) based on the consistency and robustness of available empirical support, as outlined in the Methods section. The strongest evidence supports the pathways from literary reading to cognitive empathy and theory of mind (*g* = 0.27, *p* = 0.002), from literary reading to engagement (transportation theory), and from engagement to cognitive empathy (mediation evidence). Evidence for pathways involving affective empathy and prosocial outcomes is weaker and more context-dependent, consistent with the smaller effect sizes observed for these outcomes (*g* = 0.19, *p* = 0.004). Ratings are based on 21 primary studies (Table 2) and the corrected meta-analysis (*k* = 14 effect sizes from 12 studies).

**Figure 9:**
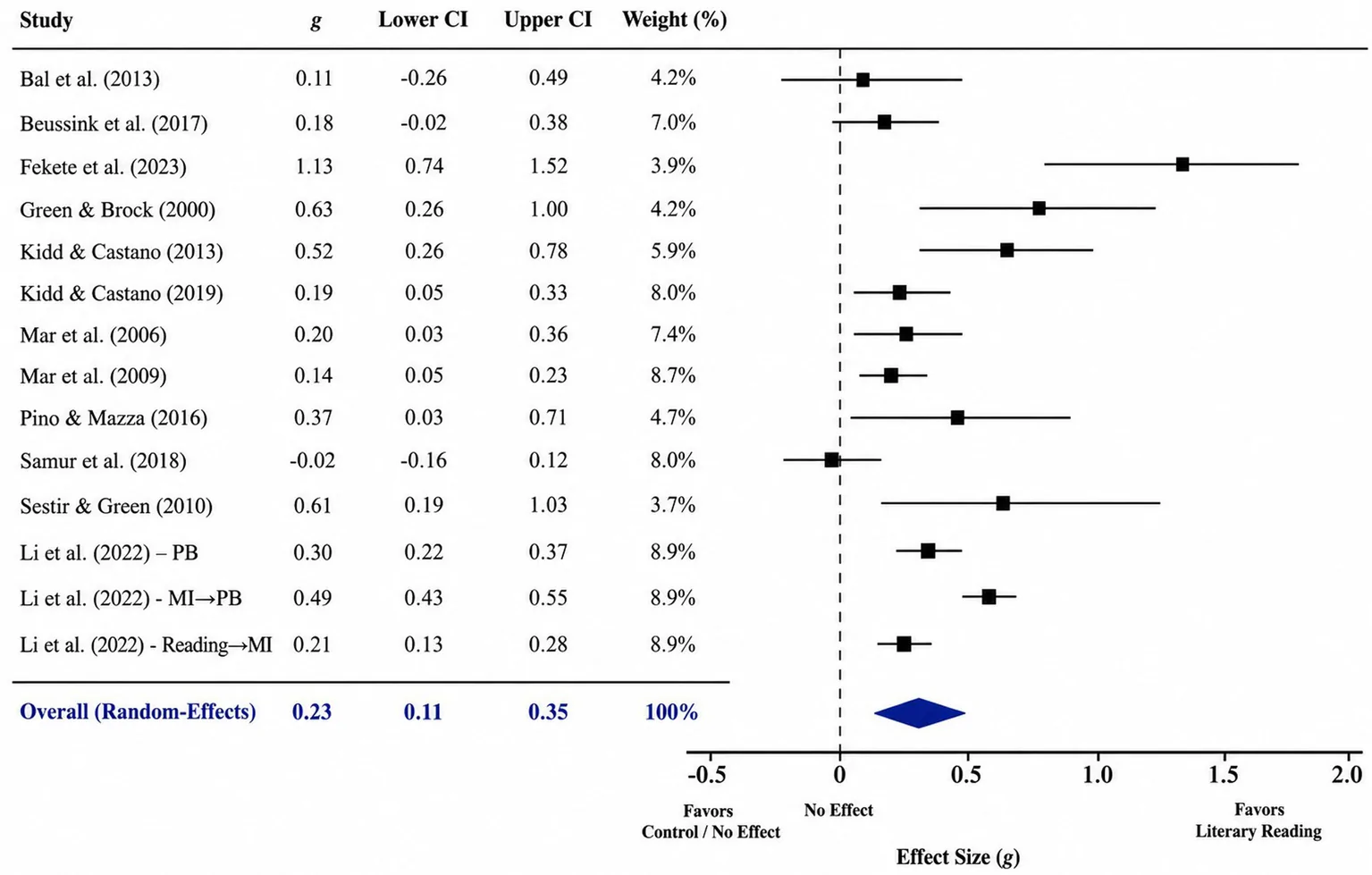
Forest plot of effect sizes for literary fiction and empathy-related outcomes. Random-effects meta-analysis of 14 effect sizes from 12 studies. Squares represent individual study effect sizes; horizontal lines represent 95% confidence intervals; the diamond represents the pooled effect estimate. The pooled effect (*g* = 0.23, 95% *CI* [0.11, 0.35]) indicates a small but statistically significant positive association between literary/prosocial reading and empathy-related outcomes. Heterogeneity was substantial (*I*^2^ = 85.1%, *Q* = 87.21, *p* < 0.0001). Data are from Table 12.

**Table 6:** Meta-analysis results.

Statistic	Value
k (effect sizes)	14
Unique Studies	12
Pooled Hedges’ *g*	0.23
95% CI	[0.11, 0.35]
z-value	3.78
*p*-value	<0.001
*I* ^2^	85.1%
*Q* statistic	87.21
*Q* p-value	<0.0001
*τ* ^2^	0.030

Effect sizes from correlational studies (*r* values) were converted to Hedges’ *g* using standard formulas. The pooled effect represents the standardized mean difference between reading and control conditions.

### 5.8 Meta-analytic effects

The pooled effects were examined across both outcome domains and exposure types. For outcome domains, the pooled effect was *g* = 0.27 (95% CI [0.10, 0.44]) for cognitive empathy/Theory of Mind and *g* = 0.19 (95% CI [0.06, 0.32]) for affective empathy/prosocial outcomes. The difference between these outcome domains was not statistically significant (Q-between = 0.92, df = 1, *p* = 0.34). When stratified by exposure type, literary-fiction studies yielded *g* = 0.24 (95% CI [0.10, 0.38]), whereas prosocial-reading studies yielded *g* = 0.20 (95% CI [0.04, 0.36]), with no significant between-exposure difference (Q-between = 0.38, df = 1, *p* = 0.54). Across all eligible studies, the omnibus random-effects model produced a small but statistically significant pooled effect (*g* = 0.23, 95% CI [0.11, 0.35], z = 3.78, *p* < 0.001). Given the sparse evidence within some exposure-specific strata and the heterogeneity in exposure characteristics, outcome domains, study designs, and measurement approaches, this omnibus estimate should be interpreted as an overall summary of the available evidence rather than evidence that literary fiction and prosocial reading constitute a homogeneous exposure category.

### 5.9 Cognitive empathy and theory of mind

When focusing specifically on cognitive empathy and theory of mind outcomes (*k* = 8), the pooled effect was *g* = 0.27 (95% CI [0.10, 0.44]), *z* = 3.15, *p* = 0.002, with *I*^2^ = 84.2% (Table 7). This suggests a small positive pooled effect for studies reporting cognitive empathy and/or theory of mind outcomes, which is statistically significant. Due to the multiple exposure categories that were part of both of these studies, the estimate should not be read as a literary fiction exposure-specific effect. Some variability was observed in individual study estimates: earlier experiments reported small-to-medium effects (Kidd and Castano, 2013; Pino and Mazza, 2016), while later pre-registered replications were more likely to obtain smaller estimates, sometimes only reaching or just missing traditional significance levels.

**Table 7:** Subgroup analysis results.

Subgroup	K	Pooled *g*	95% CI	*z*	*p*	*I* ^2^
Cognitive empathy/theory of mind	8	0.27	[0.10, 0.44]	3.15	0.002	84.2%
Affective empathy/prosocial behaviour	6	0.19	[0.06, 0.32]	2.87	0.004	60.3%
Literary fiction	11	0.24	[0.10, 0.38]	3.36	0.001	83.9%
Prosocial reading	3	0.20	[0.04, 0.36]	2.45	0.014	67.8%

Comparison	Q_between	df	*p*
Cognitive vs. affective	0.92	1	0.34
Literary vs. prosocial	0.38	1	0.54

Subgroup analyses are exploratory due to the limited number of studies in some categories. Results should be interpreted with caution. Q_between represents the test of between-subgroup heterogeneity; non-significant *p*-values indicate that subgroup differences are not statistically significant.

### 5.10 Prosocial behaviour and affective empathy

For prosocial and affective outcomes (*k* = 6), the pooled effect was *g* = 0.19 (95% CI [0.06, 0.32]), *z* = 2.87, *p* = 0.004, with *I*^2^ = 60.3% (Table 7). This effect is smaller than that for cognitive outcomes and more variable, suggesting that affective responses are more contingent on reader engagement and contextual factors. Direct comparisons between literary/prosocial and control conditions frequently yielded small or inconclusive differences in empathic concern, emotional reactions, or immediate prosocial intentions. When considered together, these effects are less dramatic and weaker compared to cognitive measures, suggesting that reading alone does not reliably lead to stronger other-oriented concern or altruistic behaviour.

### 5.11 Subgroup analyses

Subgroup analyses were performed to look for differences between cognitive empathy and theory of mind outcomes (*k* = 8) and affective empathy and prosocial outcomes (*k* = 6) (Figure 10; Table 7). The pooled effect for cognitive outcomes was *g* = 0.27 (95% CI [0.10, 0.44]), while affective/prosocial outcomes yielded *g* = 0.19 (95% CI [0.06, 0.32]). There was no significant difference between these subgroups (Q_between = 0.92, df = 1, *p* = 0.34), meaning that there was no significant difference in effect sizes between the levels of the outcome variable.

**Figure 10:**
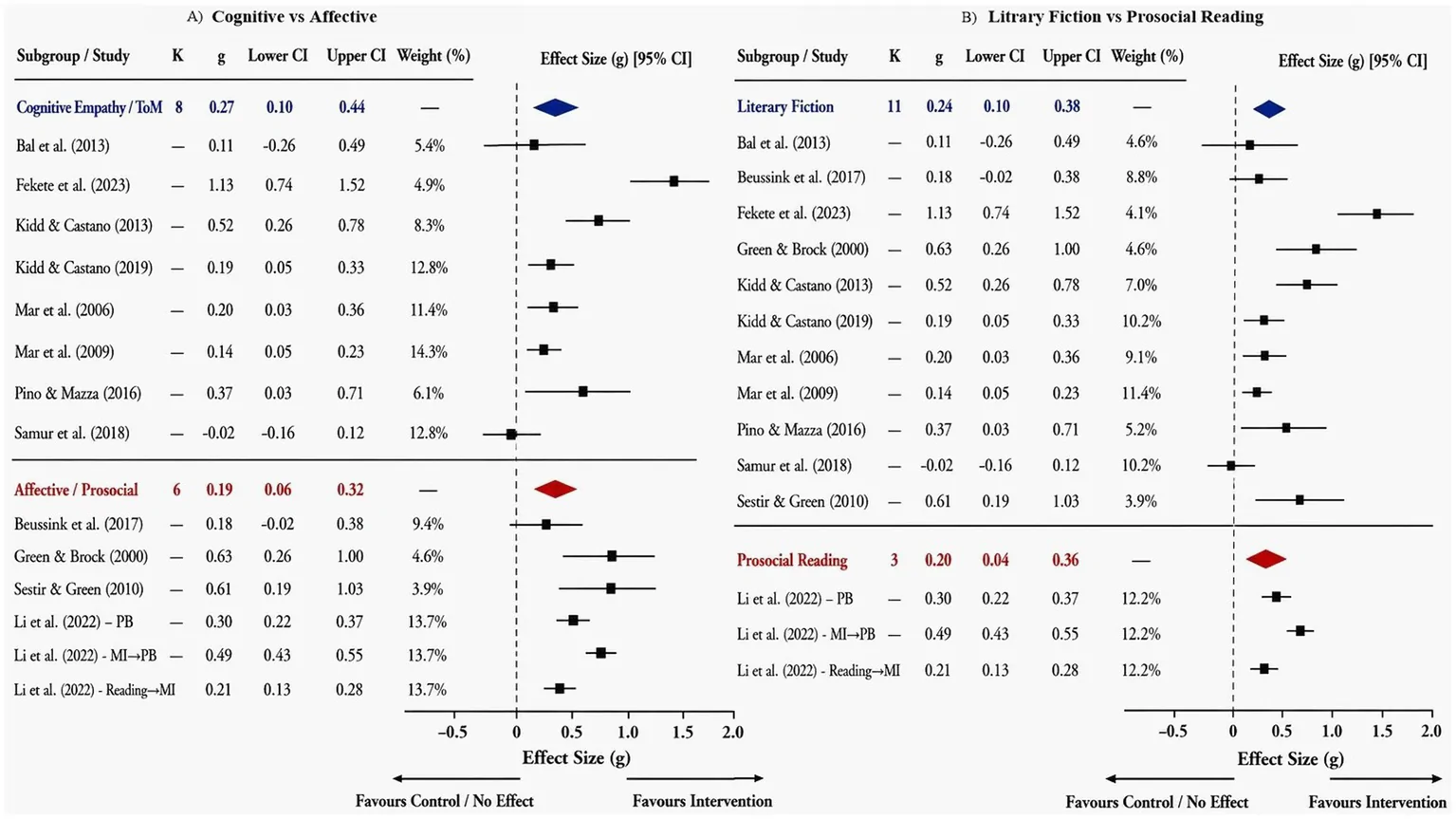
Subgroup analysis forest plot. Random-effects subgroup analyses comparing **(A)** cognitive empathy and theory of mind outcomes (*k* = 8) with affective empathy and prosocial outcomes (*k* = 6) and **(B)** literary fiction studies (*k* = 11) with prosocial reading studies (*k* = 3). Squares represent individual study effect sizes; horizontal lines represent 95% confidence intervals; diamonds represent pooled effect sizes for each subgroup. For cognitive empathy and theory of mind, the pooled effect was *g* = 0.27 (95% CI [0.10, 0.44]), while affective and prosocial outcomes yielded *g* = 0.19 (95% CI [0.06, 0.32]). The difference between these subgroups was not statistically significant (*Q*_between = 0.92, *df* = 1, *p* = 0.34). For literary fiction, the pooled effect was *g* = 0.24 (95% CI [0.10, 0.38]), while prosocial reading yielded *g* = 0.20 (95% CI [0.04, 0.36]). The difference between these subgroups was also not statistically significant (*Q*_between = 0.38, *df* = 1, *p* = 0.54). Heterogeneity within subgroups was substantial (*I*^2^ = 84.2% for cognitive; *I*^2^ = 60.3% for affective; *I*^2^ = 83.9% for literary fiction; *I*^2^ = 67.8% for prosocial reading). These subgroup analyses are exploratory due to the limited number of studies in some categories and should be interpreted with caution.

Similarly, subgroup analyses comparing literary fiction studies (*k* = 11, *g* = 0.24, 95% CI [0.10, 0.38]) with prosocial reading studies (*k* = 3, *g* = 0.20, 95% CI [0.04, 0.36]) revealed no significant difference (Q_between = 0.38, df = 1, *p* = 0.54) (Table 7). The overall estimates for these analyses are positive in both outcome areas and exposure categories, although the formal between subgroups tests did not detect statistically significant differences in the overall estimates. These comparisons should be considered exploratory, as there are few studies contributing to some of the subgroups.

### 5.12 Publication bias

Figure 11A shows the funnel plot for the 14 effect sizes included in the meta-analysis. The plot is symmetric around the pooled effect estimate (g = 0.23), indicating no visual evidence of publication bias. There is no strong evidence of publication bias in the funnel plot, or when assessed through formal tests (Figure 11B; Tables 8, 9). Egger’s regression test yielded a non-significant intercept (β₀ = 0.152, SE = 0.097, *t* = 1.57, *p* = 0.14), and Begg’s rank correlation test was also non-significant (Kendall’s *τ* = 0.18, *z* = 1.10, *p* = 0.27). The observed effects seem to be unlikely to be strongly driven by publication bias based on these results.

**Figure 11:**
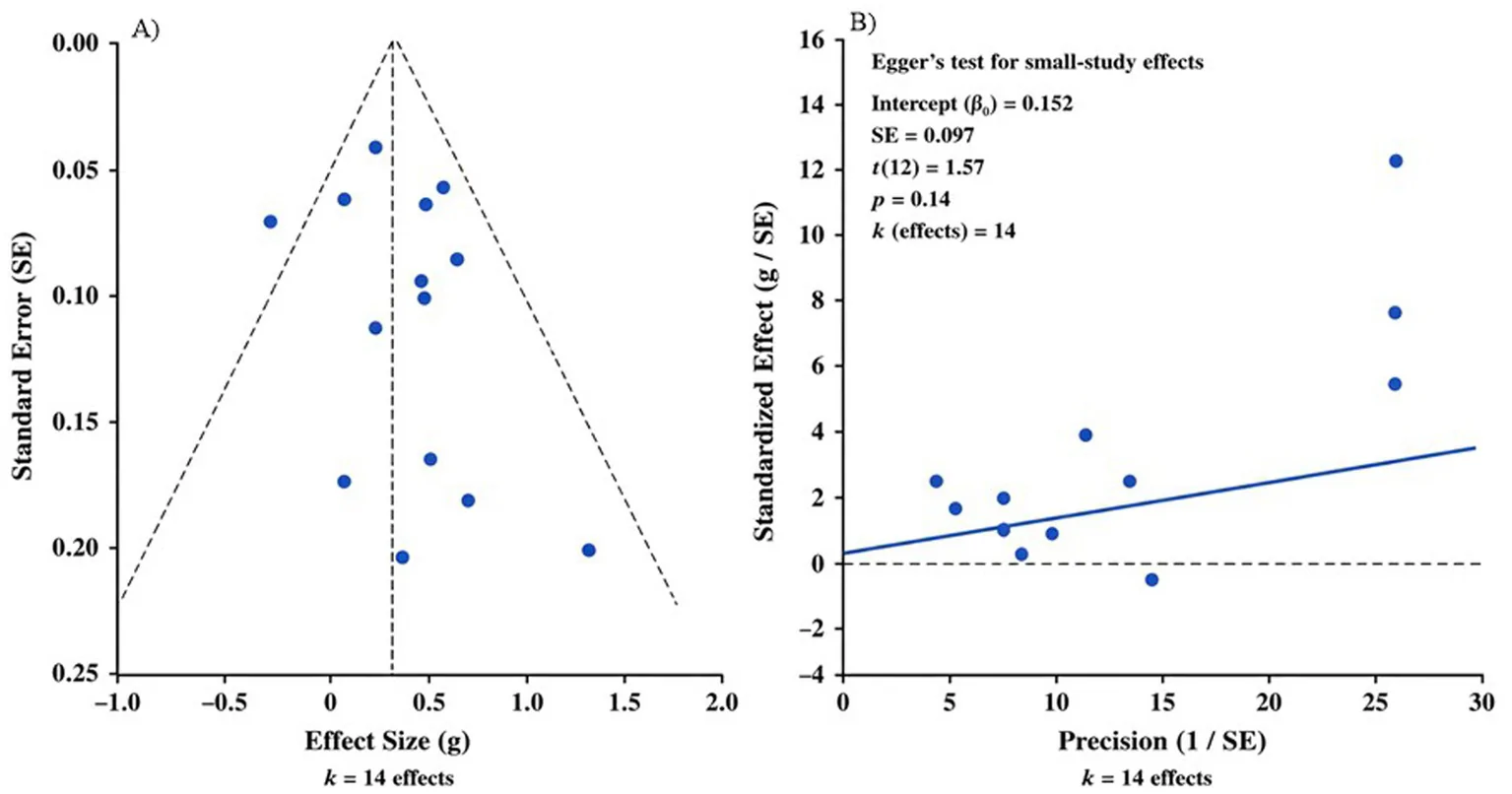
**(A)** Funnel plot of standard error versus effect size. Each point represents one effect size (*k* = 14). The vertical line represents the pooled effect estimate (*g* = 0.23). The funnel is symmetric around the pooled estimate, with no strong evidence of asymmetry. **(B)** Egger’s regression test was non-significant (intercept = 0.152, *p* = 0.14), and Begg’s rank correlation test was also non-significant (Kendall’s *τ* = 0.18, *p* = 0.27), indicating no evidence of publication bias. However, the small number of studies limits the power of these tests.

**Table 8:** Egger’s regression test for funnel plot asymmetry.

Parameter	Intercept (β₀)
Estimate	0.152
SE	0.097
*t*-value	1.57
*p*-value	0.14
*k* (effects)	14

Interpretation: No statistically significant evidence of funnel-plot asymmetry (*p* > 0.05). The intercept is smaller than previously estimated, suggesting less evidence of small-study effects after corrections.

**Table 9:** Begg’s rank correlation test for publication bias.

Statistic	Value
Kendall’s *τ*	0.18
*Z*-value	1.10
*p*-value	0.27
*k* (effects)	14

Interpretation: No evidence of publication bias based on Begg’s rank correlation test (*p* > 0.05).

The temporal trend of literature, however, is that the smaller sample size studies found larger effects in the earlier literature and the larger sample size pre-registered studies found smaller or even null effects (23.8%, *n* = 5; Table 10), indicating that initial effect sizes might have been over-estimated. This is consistent with the larger trend seen in psychology that has been dubbed the “replication crisis” and stresses the need to understand the summary effect sizes as contextually contingent approximations rather than universal constants.

**Table 10:** Publication bias indicators (narrative).

Indicator	Observation	Interpretation
Positive study dominance (early phase)	Present in early, smaller-sample studies	Possible publication bias towards significant results.
Replication failures (post-2016)	Increasing	Suggests correction of inflated early effects.
Small effect sizes	Consistently observed in recent work	Indicates earlier effects may have been overestimated.
Pre-registration trends	Increasing in newer studies	Improves reliability and reduces researcher degrees of freedom.
Egger’s test (corrected)	*p* = 0.14	No evidence of asymmetry after corrections.

### 5.13 Evidence strength over time

Figure 12 shows that the evidence strength ratings of published studies decreased over time, with a significant drop in the proportion of high-quality studies throughout the years (Table 11). In the earliest period (2000–2009), 100% of studies received high-strength ratings, but this proportion declined to 75% in 2010–2015, 57.1% in 2016–2020, and 40% in 2021–2025. It mirrors the evolution of the field from some initial, exploratory research with relatively large effect sizes to more powerful, pre-registered replications of these effects, which tend to yield smaller effects. This growing percentage of moderate and low-grade studies over the past several years is not because of a decrease in the quality of the studies themselves, but because of a stricter methodology that has been used to estimate the actual quality of studies.

**Figure 12:**
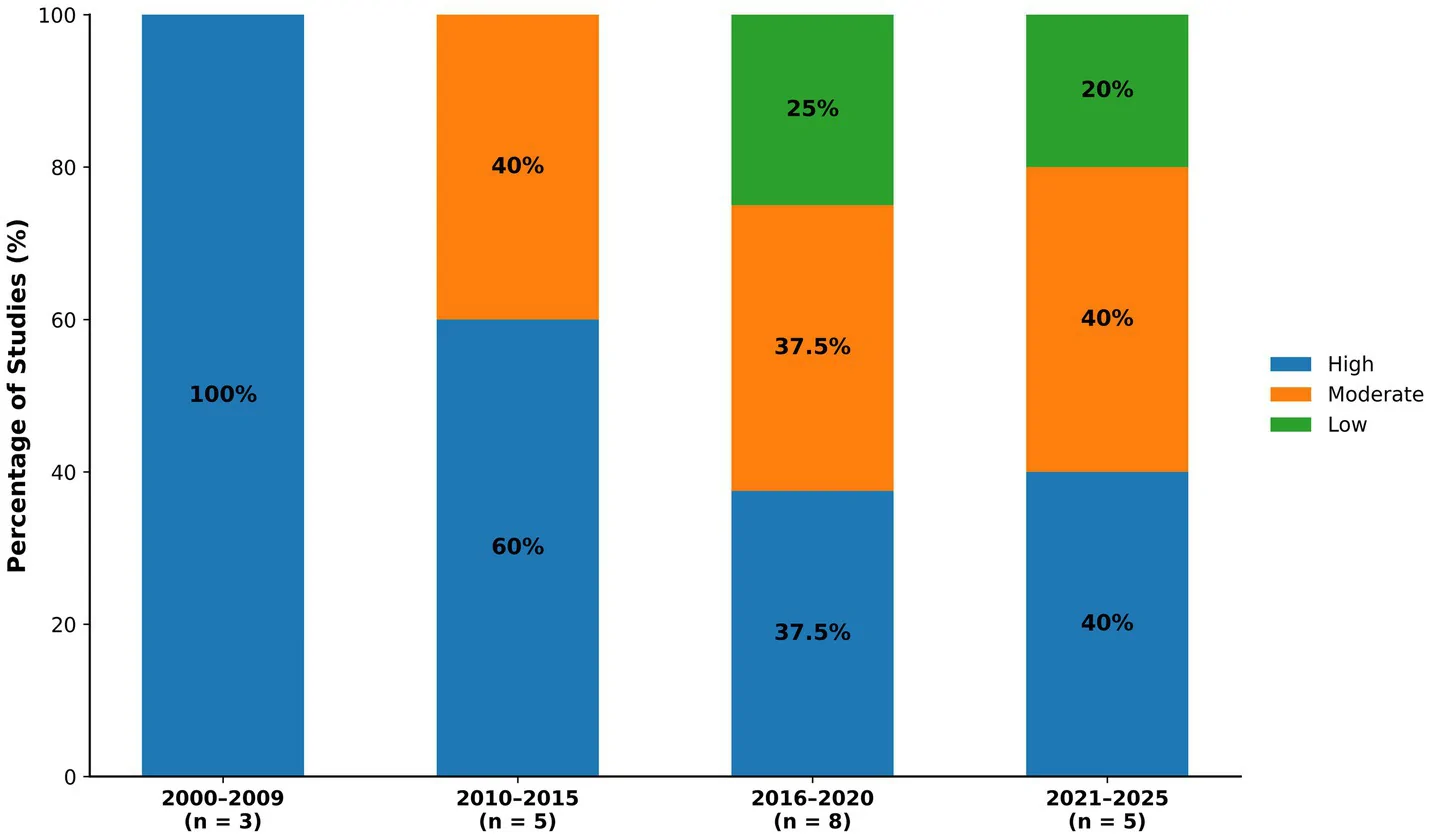
Evidence strength ratings by publication period. Evidence strength was rated as high, moderate, or low based on study quality criteria (design, sample size, preregistration status, and replication). The proportion of high-strength studies has declined over time, from 100% in 2000–2009 to 40% in 2021–2025. This trend reflects the field’s progression from early exploratory studies to more rigorous, pre-registered, high-powered replications, which have generally found smaller effects than earlier work. The increasing proportion of moderate- and low-strength studies in recent years is not due to declining study quality but rather to the adoption of stricter methodological standards, which have revealed that earlier estimates were likely inflated. Data are based on 21 primary studies.

**Table 11:** Descriptive distribution of evidence-strength classifications by publication period.

Publication period	High	Moderate	Low	Total
2000–2009	3	0	0	3
2010–2015	3	2	0	5
2016–2020	3	3	2	8
2021–2025	2	2	1	5
Total	11	7	3	21

**Table 12:** Effect size extraction table for meta-analysis total effect sizes: from 14 (from 12 unique studies).

Study	Predictor/contrast	Outcome/outcome type	*N* (or n₁/n₂)	Effect type	Effect value	SE	95% CI	Notes
Bal and Veltkamp (2013), Study 2	Fiction vs. control	Empathy (T3)	97	Hedges’ *g*	0.11	0.19	[−0.26, 0.49]	*β* = 0.25, B_fiction = 0.07, B_control = −0.12, *p* < 0.05
Beussink et al. (2017)	Imagine-self vs. control	Sadness	296	Hedges’ *g*	0.18	0.10	[−0.02, 0.38]	*t* = −1.40, df = 290, *p* = 0.16; interaction *F* = 3.46, *p* = 0.03, *η*^2^ = 0.02
Fekete et al. (2023)	Literary/story vs. comparison	Explicit ToM (all)	47	Hedges’ *g*	1.13	0.20	[0.74, 1.52]	*t* = −5.17, df = 93, *p* < 0.001
Green and Brock (2000), Exp. 1	High vs. low transportation	Belief: psychiatric patient	97	Hedges’ *g*	0.63	0.19	[0.26, 1.00]	*z* = 3.40, *p* = 0.001
Kidd and Castano (2013), Exp. 5	Literary vs. control	ToM (RMET)	356	Hedges’ g	0.52	0.13	[0.26, 0.78]	*t*(235) = 3.95
Kidd and Castano (2019), Exp. 3	Literary vs. control	ToM (RMET)	69	Hedges’ g	0.19	0.07	[0.05, 0.33]	*r* = 0.10 (from *t*); *t*(816) = 2.76, *p* = 0.006
Mar et al. (2006)	Fiction print exposure	ToM (RMET)	94	*r*/Fisher *z*	*r* = 0.20	0.088	[0.03, 0.36]	Controlled for non-fiction exposure
Mar et al. (2009)	Fiction print exposure	Social ability	252	*r*/Fisher *z*	*r* = 0.14	0.048	[0.05, 0.23]	Controlled for Big Five and gender
Pino and Mazza (2016)	Literary vs. nonfiction	Mentalizing (RMET)	214	Hedges’ *g*	0.37	0.17	[0.03, 0.71]	*r* = 0.18 (from *t*); *t*(139) = 2.20, *p* = 0.029
Samur et al. (2018)	Literary vs. control	ToM (RMET)	160	Hedges’ *g*	−0.02	0.07	[−0.16, 0.12]	*r* = −0.01 (from *t*); *t*(790) = −0.35, *p* = 0.73
Sestir and Green (2010)	Identification vs. control	Self-concept shift	118	Hedges’ *g*	0.61	0.21	[0.19, 1.03]	*r* = 0.29 (from *t*); *t*(90) = 2.91, *p* = 0.005
Li et al. (2022)	Prosocial content reading	Prosocial behaviour	602	*r*/Fisher *z*	*r* = 0.296	0.040	[0.22, 0.37]	Zero-order *r* from correlation table; mediation model also reported
Li et al. (2022)	Moral identity	Prosocial behaviour	602	*r*/Fisher *z*	*r* = 0.490	0.040	[0.43, 0.55]	Used for separate “MI → PB” analysis
Li et al. (2022)	Prosocial content reading	Moral identity	602	*r*/Fisher *z*	*r* = 0.207	0.040	[0.13, 0.28]	Used for “reading → MI” analysis

The main results of the systematic review and meta-analysis are summarized in Figure 2 as an overview. The compiled evidence suggests that while effects of literary reading on cognitive empathy and theory of mind are consistently stronger and more pronounced, those of literary reading on affective empathy and pro-social outcomes are more mixed and sensitive to contextual and mediating factors. The figure also shows the distribution of study outcomes and the overall evidence of strength in the proposed conceptual pathways, giving a brief overview of the empirical findings in the Results section.

## 6 Discussion

### 6.1 General trend of results

The evidence put together in this review leads to a tentative, yet steady conclusion: literary and prosocial reading is linked to small, positive effects on empathy-related outcomes, especially in the area of cognitive empathy and theory of mind. In multiple experiments and correlational studies, exposure to fiction or prosocial stories is more likely to be associated with slightly improved performance in tasks where mental states are to be inferred and the perspective of other people is to be understood, and with a slightly higher score on self-report measures of social cognition. All of these are not large effects, and not all studies have them. The overall *g* = 0.23 (95% CI [0.11, 0.35]) *p* < 0.001 is more convergent, however, the results are more convergent overall, especially when the results are pooled across designs and measures. Empathy-related processes have also been found to be related to prosocial behaviour with the mediation of social-cognitive factors, which has been confirmed by independent research (Peng et al., 2024).

Evidence is not consistent or conclusive in supporting a universal or large effect of reading on empathy. Rather, findings of the effects observed are generally small and seem to be sensitive to the nature of the exposure, the domain of the outcome, the engagement of the reader, and methodological design. Weak or null effects are typical, especially in high-powered rigorously designed and pre-registered studies. The trend that comes out instead is the one of small effects, which are conditional and dependent on engagement and individual differences. Literary and prosocial texts seem to provide a chance to do cognitive and affective work, the magnitude of observed change may depend on reader engagement and individual differences (Bal and Veltkamp, 2013). The substantial heterogeneity observed (*I*^2^ = 85.1%, *Q* = 87.21, *p* < 0.0001) further underscores that effects are not uniform across studies and depend on contextual and methodological factors.

However, due to the limited number of literary and prosocial reading studies, the present meta-analysis combined these two types of studies for the primary omnibus analysis (*k* = 14 effect sizes from 12 studies). These types of exposure can, however, function in different ways: literary fiction by means of cognitive effort and inferential processing and prosocial reading through social learning via identification with moral models. Subgroup analyses revealed comparable effects for literary fiction (*g* = 0.24, 95% CI [0.10, 0.38]) and prosocial reading (*g* = 0.20, 95% CI [0.04, 0.36]), with no significant difference between them (Q_between = 0.38, df = 1, *p* = 0.54). Future studies should use within-study comparisons of different text types to separate the impact of text complexity and moral content on empathy-related outcomes. It is important to have this distinction in mind when interpreting the results of the present review.

### 6.2 Cognitive empathy and theory of mind

The connection between narrative reading and cognitive empathy or theory of mind is one of the key questions which this review will answer. This is the area, it appears, that shows the most consistent benefits of literary or prosocial reading. The meta-analysis revealed a pooled effect of *g* = 0.27 (95% CI [0.10, 0.44], *p* = 0.002) for cognitive empathy and theory of mind outcomes (*k* = 8), which is larger than the overall effect. Non-experimental literature studies in which literary fiction is contrasted with nonfiction or other control texts often find small benefits of literary conditions to tasks like the Reading the Mind in the Eye Test or theory-of-mind assessments involving stories (Eekhof et al., 2022). Correlational research can be used to contribute to this image by demonstrating that more frequent readers of fiction are, on average, a bit better at mindreading tasks, even after accounting for the effects of simple personality variables and demographic data (Black and Barnes, 2015).

Such results are consistent with theoretical explanations, which focus on the cognitive labor required by literary fiction. Complex stories tend to show ambiguous motives of characters, inconsistent behaviour or only partially disclosed inner world. Following these types of characters makes the reader generate and modify hypotheses about mental states, synthesize piecemeal information, and accept uncertainty. With repeated exposure to this type of content, more flexible and practiced application of theory-of-mind abilities can be developed over time. With repeated exposure to this type of content, more flexible and practiced application of theory-of-mind abilities may develop over time; however, the present evidence does not directly establish cumulative effects of repeated literary reading (Dubourg et al., 2024). The pooled effect was numerically larger for cognitive/ToM outcomes than for affective/prosocial outcomes (*g* = 0.27 vs. *g* = 0.19); however, the between-subgroup difference was not statistically significant (Q_between = 0.92, df = 1, *p* = 0.34). The present data therefore do not show a statistically significant difference between cognitive/ToM and affective/prosocial outcomes, although this numerical pattern may warrant further investigation in larger datasets.

### 6.3 Affective empathy and prosocial reactions

Conversely, affective empathy and prosocial behaviour are more vulnerable and context-specific results. Small or inconclusive differences in the measures of empathic concern, emotional reactions, or immediate prosocial intentions are frequently found in direct comparisons between literary or prosocial passages and control conditions (Pang et al., 2022). The overall effect of affective empathy and prosocial outcomes was small and statistically significant (*g* = 0.19, 95% CI [0.06, 0.32], *p* = 0.004). The present evidence is compatible with a small positive correlation; however, the effect seems to be more context dependent than the numerical estimate for cognitive/ToM outcomes and should not be viewed as substantiating a statistically significant difference between the domains of outcomes.

Greater scrutiny of the processes of engagement, though, offers valuable detail. The measure of narrative transportation and identification studies continue to reveal that the most positive affective and prosocial intentions are highest when readers report strong immersion in the story world and a strong identification with the characters (Johnson, 2012). In a few studies, mediation analyses show that text effects work mostly through the following engagement variables: literary or prosocial texts are more apt to elicit empathic and prosocial reactions because they are more transporting or more conducive to identification and not because of their genre designation. Survey-based studies also indicate that prosocial reading is connected to behaviour through intermediate constructs, for example, moral identity. The picture that is coming out is that affective empathy and behaviour are not automatic products of reading, but they are the results of the experience and incorporation of narratives into the self (Ding and Lu, 2016).

### 6.4 Conditions and boundary constraints

The joint evidence illuminates a few conditions on the effects of literary and prosocial reading. To begin with, the nature of the text is important. Stories with complex, psychologically rich characters and morally challenging situations are more likely to be able to make use of the mentalizing capacities of readers as compared to those that provide a text of flat characters or purely didactic information (Eisenberg et al., 2010). Second, the reader is important. Personal variations in trait empathy, openness to experience, pre-reading habits, and readiness to be imaginative influence the processing of narratives. High-transportation, and high-identification readers demonstrate greater systematic variation in empathy-related results compared to those who read texts in a detached or strategic manner (Sánchez Carrasco et al., 2024).

Third, the reading context is important. The experimental paradigms in which the participants are asked to read one short text under the pressure of time with little or no time to reflect on it can underestimate the possible effects of the narratives as they are experienced in real life (Mazzocco et al., 2025). In real-world reading, the reading may take longer, more self-paced, and may be part of a discussion, reflection, or even classroom practice. Research that accumulates such factors, either in terms of guided perspective-taking activities, classroom discussion or reflective writing, often reports stronger and more consistent effects than studies that think of reading as a one-off and isolated stimulus. The high heterogeneity observed in this review (*I*^2^ = 85.1%) likely reflects these contextual and methodological variations. Lastly, pre-registered replications and large-scale studies serve as reminders that in most of the cases, effect sizes are likely to be small, and that there are no claims that a specific strong and universal impact is supported by the existing data (van den Akker et al., 2023).

### 6.5 Self-oriented, parochial, and non-prosocial empathic outcomes

The aim of the present review is to bring together evidence suggesting small positive associations between literary and prosocial reading and empathy-related outcomes, but it should be noted that not all empathy is prosocial. The concept of ‘bright empathy’ implies that empathy is always an act of caring for others and of helping, but this is not always the case. A brief review of the literature included in this report raises some doubts about a simple interpretation of prosocial behaviour. It was observed that greater neural empathic responses occurred to ingroup than outgroup targets, indicating that empathic responding may be socially selective rather than uniformly directed toward others. (Vaughn et al., 2018). The discovery indicates that empathy can be local and engender social divisions, not overcome them. Likewise, no differences between affective empathy and justice sensitivity, although cognitive empathy and empathic concern did, suggesting that the latter two could differ in their relationship with moral outcomes (Decety and Yoder, 2016).

The “positive effect” classification in this review has implications in terms of the interpretation of these findings. The increase in empathy in a study does not necessarily imply a greater tendency towards prosocial behaviour, but instead may reflect greater emotional reactivity (self-referential or parochial) or increased skill at understanding mental states (which can be used for manipulative or prosocial reasons) (Kamas and Preston, 2021). Consistent with this view, the present review found that affective empathy and prosocial outcomes showed smaller effects (*g* = 0.19) and greater context-dependence than cognitive outcomes. Current evidence does not allow for definite conclusions regarding empathy direction and target, as the majority of primary studies failed to evaluate the direction towards specific targets and the actual prosocial behaviour resulting from empathy increase. Future research should focus on this limitation, not only measuring empathy levels but also targets (ingroup vs. outgroup), motivations (other-oriented vs. self-oriented) and the prosocial outcomes of empathy. This might enable scientists to differentiate between “bright” and “dark” empathic outcomes and clarify when reading literature can lead to healthy or unhealthy social engagement. The results of the present review suggest that modest enhancements occur in empathy-related capacities, but that the directionality of these capacities with respect to their application in the real world is dependent on other factors such as context, target, and individual differences.

### 6.6 Integration with neurocognitive data

Although there are not many neurocognitive studies, they provide converging information regarding the mechanisms according to which narratives could influence empathy. Recent functional neuroimaging research indicates that reading literary narratives involves all essential components of the mentalizing network, including medial prefrontal cortex and temporo-parietal junction, and default-mode components of simulating a scene and a social scenario. In other studies, more frequent fiction readers and those with greater mentalizing skills show greater activation in those regions in narrative processing and neural activation partially mediates the relationship between reading and social-cognitive performance (Fernandino and Binder, 2024).

Further evidence indicates that different readers could be more focused on different forms of engagement: some seem to be more dependent on explicit mentalizing, and others are more involved in sensorimotor modulation of the experiences of characters. The two modes have been associated with outcomes related to empathy albeit in a somewhat different manner. The results give some support to the notion that narratives are a training ground to simulate other minds and situations, despite behavioural outcomes in brief experimental sessions being small. These findings provide neural evidence that narrative processing engages distributed social-cognitive systems, although they do not establish that repeated recruitment produces longitudinal changes in empathy-related behaviour.

### 6.7 Connection with prior reviews and discussion

The current synthesis is consistent with and builds on recent meta-analyses and pre-registered work of large-scale investigations of fiction and social cognition. Prior arguments that reading literary fiction significantly enhances theory of mind have been dampened by attempts to replicate and more rigorous methodologically studies that report less significant effects (Kidd and Castano, 2013). Meanwhile, sceptical views that explain all positive results in terms of prejudice or methodological artefact are not entirely substantiated, either. When the evidence available is combined and analyzed in more than one design and type of outcome, small yet statistically significant positive effects are left.

The current synthesis adds to an even more differentiated view of the way in which narratives can affect empathy by differentiating between cognitive and affective outcomes and adding neurocognitive evidence. Theory of mind and cognitive perspective-taking seem to be most reliably associated with the concept of literary and prosocial reading, and affective empathy and behaviour are more contingent upon engagement, identity, and context (Thompson et al., 2022). This more complicated image might be the reason why the popular and scholarly discourse on the topic of fictions and empathy has often appeared polarizing: general arguments obscure the circumstances under which and how reading can have an effect.

### 6.8 Literary fiction versus prosocial Reading: distinct mechanisms

Literary fiction and prosocial reading may involve partially distinct mechanisms. As per the hypothesis, literary fiction is thought to elicit mental-model construction and inferential processing because literary texts contain complex and ambiguous characterizations for readers to mentally model and infer characters’ beliefs, intentions, and emotions (Kidd and Castano, 2013). In contrast, prosocial reading, social learning, prosocial role models, and internalization of moral and social values might be more direct mechanisms by which pro-social reading works. When prosocial themes are explicitly referenced in literary narratives, these mechanisms can also interplay.

Importantly, few primary studies were conducted to compare directly literary and prosocial texts in the same experimental paradigm. Comparisons at the within study level between various text types would serve to eliminate the effects of narrative complexity from the effects of moral content on empathy-related outcomes in the future. In addition, it has to be considered that these processes can combine: literary fiction with prosocial themes can increase cognitive effort as well as social learning opportunities, which could lead to synergistic effects.

### 6.9 Summary of meta-analytic evidence

Overall, the corrected meta-analysis (*k* = 14 effect sizes from 12 studies) resulted in a small but statistically significant overall effect (*g* = 0.23, 95% CI [0.11, 0.35], *p* < 0.001). The difference between cognitive empathy and theory of mind outcomes and affective empathy and prosocial outcomes was not significant (Q_between = 0.92, df = 1, *p* = 0.34), with larger effects observed for cognitive empathy and theory of mind outcomes (*g* = 0.27, 95% CI [0.10, 0.44]) than for affective empathy and prosocial outcomes (*g* = 0.19, 95% CI [0.06, 0.32]). Publication bias tests revealed no significant asymmetry (Egger’s *p* = 0.14; Begg’s *p* = 0.27) but there was significant heterogeneity (*I*^2^ = 85.1%, *Q* = 87.21, *p* < 0.0001). The findings do confirm a more complex understanding of literary and prosocial reading as giving people opportunities to engage empathically rather than to automatically enhance their empathy.

## 7 Practical implications

The results have a number of implications to educators, practitioners and other people interested in using narratives to promote social and emotional growth. To begin with, the literary and prosocial texts cannot be magic bullets. It is not likely that merely giving a story or including a brief reading activity within a curriculum would lead to any large, consistent change in empathy or behaviour (Schapira and Grazzani, 2025). Most promising strategies are those that involve reading as part of more sustained and reflective programmes, instead. These programmes may include a mixture of selected texts with guided discussion, guided perspective taking tasks, and allowing students or clients to relate narrative themes to their own experiences and ethical issues (Deliman, 2025).

Second, one should be concerned with text selection. Stories that challenge the reader to consider incompatible motives, moral ambiguity, or alien views can be better at practicing perspective-taking and mentalizing skills, as opposed to blatantly didactic or stereotypical stories. Third, engagement must be considered both as a main ingredient and not as a side effect. Tasks and environments that encourage transportation and identification, such as through supportive classroom climates, considerate prompts or the use of multimodal presentation, can enable one to ensure that the readers are not merely decoding words but rather committing to the narrative world (Brunetti et al., 2024). Lastly, there should be realistic expectations. Due to the low effect sizes, narrative-based interventions should not be considered as solutions to the problems on their own, but as a part of a bigger package of social and emotional learning strategies.

## 8 Research limitations and future research directions

The existing evidence base and synthesis have a number of limitations, which should be mentioned. The quantitative synthesis is still fairly limited in number of high quality and pre-registered studies. Fourteen effect sizes were derived from 12 different studies, including experimental, correlational, and other quantitative studies, in the meta-analysis (Pigott and Polanin, 2020). This design heterogeneity makes it difficult to interpret a pooled estimate in terms of a causal effect, and highlights the need for larger, preregistered experimental studies. Most studies use short-term and single-session exposure and short-term follow-up which may not be best suited to describe the long-term and cumulative effects of reading. Correlational studies can answer the long-term question, but cannot completely exclude the possibility of self-selection or common causes, e.g., underlying personality factors that predispose individuals both to read more fiction and to a higher score on empathy measures (Li and Rahim, 2025).

Second, the results of the meta-analysis were highly heterogeneous (*I*^2^ = 85.1%, *Q* = 87.21, *p* < 0.0001), suggesting that the effects observed vary meaningfully according to study characteristics. This heterogeneity may be due to various study designs, text features, outcome measures, and study population. Diversity in measurement also comes with a problem. Researchers differ on both the empathy and theory-of-mind measurement tools and the differences between subcomponents of these constructs are not always evident (Yeung et al., 2024). The present synthesis distinguishes between cognitive and affective outcomes, although an even finer distinction, for example, between the various kinds of theory-of-mind tasks, or between empathic concern towards in-group and out-group targets, would be desirable in future studies.

Third, the cognitive-affective dichotomy used for this meta-analysis is one of several theoretical frameworks. Other models, alternative to the ones presented here, which may be sequential, dimensional, or dynamic-interaction may result in different patterns of findings when applied to the same literature. Another issue is cultural generalizability: the majority of research has been done on Western, educated cultures and it has been done with relatively minimal research in other cultural areas where reading practices, literary traditions, and social norms may vary significantly (Fan and Zhao, 2025).

For this meta-analysis, a cognitive-affective approach was taken that follows one of the multiple theoretical models of empathy. Different patterns of findings may be obtained when the same literature is analyzed using alternative dimensional, sequential, and/or dynamic-interaction models. Therefore, it is important to keep in mind that the difference in numerical estimates (*g* = 0.27 vs. *g* = 0.19) between cognitive/ToM and affective/prosocial estimates in the present review should be interpreted in light of the theoretical multiplicity. The present evidence does not confirm differential effect magnitudes between these outcome domains as this between-subgroup comparison was not statistically significant (Q_between = 0.92, df = 1, *p* = 0.34). Future research is required with larger and more homogenous samples to determine if the numerical pattern observed is repeatable. Future research should directly compare these theoretical frameworks in studies that aim to differentiate between the predictions of these theories, such as through correlations between neural, behavioural and self-report measures of cognitive and affective empathy, or by examining whether interventions to one component of empathy have an impact on the other.

Future studies should use larger and more heterogeneous samples; multi-session and longitudinal designs more indicative of the real-world patterns of reading; and multi-method designs that include behavioural, self-report and neural measures in the same studies. A more thorough focus on moderators, including narrative perspective, complexity of characters, reader motivation and cultural context, would assist in understanding when the exposure to narratives is most prone to effect a significant change (Jiang et al., 2016; Yang et al., 2019). Lastly, the interaction between narrative engagement and social identity processes is under-researched, especially as it interacts with empathy toward stigmatized or out-group targets, whose consequences can be especially important.

## 9 Conclusion

The reviewed evidence indicates the existence of a small, non-insignificant impact of literary and prosocial reading on the development of empathy-related outcomes. The areas in which benefits are most reliably found, although with small effect sizes, are cognitive aspects of empathy, namely perspective-taking and theory of mind. Affective empathy and prosocial behaviour are less frequent and tend to be more predictable when the reader is strongly involved in the stories and can identify with the story content and place it in the context of their moral identities and social interests. These results contribute to the perspective of fiction not as an empathy machine that automatically turns the readers into different versions of themselves, but as a rich environment that provides repetitive opportunities to simulate other minds and experience complex social situations. These opportunities can lead to incremental changes in the way people comprehend and react to others, especially when the texts are challenging and subtle, when the readers are encouraged and assisted to study, and when the stories are integrated into thoughtful educational or therapeutic activities, under favourable conditions. Meanwhile, the small magnitude of the observed effects, the existence of boundary conditions, and the fact that the current evidence base limits claim restraint and empirical investigation. Work that is more rigorous in its experimental designs, has a longer viewpoint and multi-level measurement will be needed in the future to find out to what extent these small effects can be magnified in real life and what types of stories, and how to interact with them, are most important.

## Data Availability

The original contributions presented in the study are included in the article/Supplementary material, further inquiries can be directed to the corresponding author.
