# Supplementary material for: Empathy and perspective-taking in literary fiction: a systematic review and meta-analysis of psychological evidence and theoretical insights
Source: Front Psychol. 2026 Sep 25;17:1865031. doi: 10.3389/fpsyg.2026.1865031 (PMC13649978; doi:10.3389/fpsyg.2026.1865031)
Supplement: Supplementary file 1 [file Supplementary_file_1.DOCX]

**Empathy and Perspective-Taking in Literary Fiction: A Systematic Review and Meta-Analysis of Psychological Evidence and Theoretical Insights**

**Figure S1.** *Systematic search and study selection process. The flowchart details the identification of records through database searching (Cochrane, Google Scholar, PubMed, and ScienceDirect), the screening of titles and abstracts, and the final inclusion of 15 studies for meta-analytic synthesis after applying specific inclusion and exclusion criteria.*
